# Can a linguistic serial founder effect originating in Africa explain the worldwide phonemic cline?

**DOI:** 10.1098/rsif.2016.0185

**Published:** 2016-04

**Authors:** Joaquim Fort, Joaquim Pérez-Losada

**Affiliations:** 1 Complex Systems Laboratory, University of Girona, Ma. Aurèlia Capmany 61, 17071 Girona, Catalonia, Spain; 2 Catalan Institution for Research and Advanced Studies (ICREA), Lluís Companys 23, 08010 Barcelona, Catalonia, Spain

**Keywords:** phonemic diversity, front propagation, random walks, founder effect

## Abstract

It has been proposed that a serial founder effect could have caused the present observed pattern of global phonemic diversity. Here we present a model that simulates the human range expansion out of Africa and the subsequent spatial linguistic dynamics until today. It does not assume copying errors, Darwinian competition, reduced contrastive possibilities or any other specific linguistic mechanism. We show that the decrease of linguistic diversity with distance (from the presumed origin of the expansion) arises under three assumptions, previously introduced by other authors: (i) an accumulation rate for phonemes; (ii) small phonemic inventories for the languages spoken before the out-of-Africa dispersal; (iii) an increase in the phonemic accumulation rate with the number of speakers per unit area. Numerical simulations show that the predictions of the model agree with the observed decrease of linguistic diversity with increasing distance from the most likely origin of the out-of-Africa dispersal. Thus, the proposal that a serial founder effect could have caused the present observed pattern of global phonemic diversity is viable, if three strong assumptions are satisfied.

## 1. Introduction

The dynamics of human populations over tens of thousands of years is substantially simpler to analyse than that of languages. Indeed, in the former case, we often have archaeological data that make it possible to trace population changes in time and space. In contrast, we lack any data about the languages spoken so long ago. For this reason, whereas radiocarbon dating has made it possible to conclude that human populations originated in Africa, a much more difficult and controversial topic is the possibility of tracing the ultimate spatio-temporal origin(s) of present human languages. Some recent approaches to this problem have been based on the differences among languages in phonemic diversity (i.e. in their number of phonemes).

Trudgill [1] noted that during the Austronesian Neolithic range expansion across the Pacific, the languages that evolved had fewer phonemes the further they were form the centre of dispersal. Later, Hay & Bauer [2] discovered an unexpected worldwide relationship, according to which languages with more speakers tend to have more phonemes. Although there are of course many exceptions to this relationship, Hay & Bauer [2] tested it statistically, and stressed that they had no explanation for it. In fact, Trudgill [1] suggested that if a language has more neighbouring languages, then its phoneme inventory size might perhaps increase by borrowing phonemes from its neighbours. However, according to a very recent statistical analysis by Creanza *et al.* [3], inventory size does not increase with the number of neighbouring languages. Thus, languages with more speakers tend to have more phonemes, but the mechanism behind this relationship remains unclear.

Perreault & Mathew [4] have proposed the possibility that some phonemic inventories may increase over time. They considered the coastal region spanning from southern India to the Malay Peninsula and denoted the population that arrived to this region (during the out-of-Africa dispersal of modern humans from Africa) as population A. This population then dispersed across Mainland Southeast Asia (population B) and also (in the opposite direction) into the Andaman Islands (population C), a landscape of small islands located in the Indian Ocean (for a map, see their figure 2). Perreault & Mathew [4] noted that, although both regions (B and C) were colonized at similar times and from the same place (region A), the languages presently spoken in region B have an average of *P*_B_ = 41.2 phonemes, which is substantially larger than the corresponding value in region C (*P*_C_ = 24). On the other hand, a maximum population of only about 5000 people for region C (the Andaman Islands) has been estimated before contact with Europeans. In contrast, the population size of region B (Mainland Southeast Asia) became much larger as the wave of advance of modern humans spread across this huge region. Perreault & Mathew [4] argued that the fact that languages with more speakers tend to have more phonemes [2] suggests that new phonemes are more likely to appear in large populations and, for this reason, they assumed that for the very large population in region B, some mechanism of phoneme accumulation might have gradually increased the phonemic inventories of the new languages arising from diversification. In contrast, for the very small population in the Andaman Islands (region C), Perreault & Mathew [4] assumed that the phonemic inventory size remained approximately constant. Owing to the latter assumption, the present value of *P*_C_ can be used as an estimation of the phonemic diversity (*P*_0_) of languages spoken in region A by the first humans that colonized regions B and C. Thus, Perreault & Mathew [4] estimated a linear phonemic accumulation rate as (*P*_B_ − *P*_0_)/(*t* − *t*_0_), with (*P*_B_ − *P*_0_) ≈ (*P*_B_ − *P*_C_), the values of *P*_B_ and *P*_C_ given above, and a time interval (*t* − *t*_0_) between 45 and 65 kyr (the time elapsed since the time *t*_0_ when region B was colonized until the present time *t*). This yields a rate between 0.26 and 0.38 phonemes kyr^−1^. As admitted by Perreault & Mathew [4], the above-mentioned assumptions have not been validated. On the other hand, even if phonemic diversity increased in region B and remained constant in region C, then this might result from the difference in their population sizes [4] or, alternatively, in other parameters, that might be important in principle (e.g. the mean number of speakers per language, the population density, the social network structure (which is possibly related to geography), etc.). Nevertheless, the idea [4] that some populations gain or accumulate phonemes over time is undoubtedly a novel approach that deserves further investigation. In this paper, we will analyse the possibility to connect it to the serial founder effect (as proposed by Atkinson [5], see below).

Present African languages have some of the largest phonemic inventories in the world. In contrast, as noted by Atkinson, languages with the smallest inventories are spoken in South America and Oceania [5]. The latter were the last regions to be occupied by modern humans after the out-of-Africa range expansion [6]. Atkinson [5] hypothesized that during the out-of-Africa human dispersal about 70 kyr ago [7], phoneme inventory size could have decreased owing to a serial founder effect. A founder or drift effect is the decrease in diversity owing to the random sampling of a subset of variants in a population. Clearly, such an effect is more important for small populations (namely, the pioneering ones during the out-of-Africa expansion). The word ‘serial’ refers to the repetition of such an effect during the out-of-Africa expansion. A serial founder effect had been previously proposed to explain the observed decrease in *genetic* diversity with distance from Africa [8,9]. Thus, although the correlation of *phonemic* diversity on distance from Africa is not as strong as that of *genetic* diversity on distance from Africa, both clines might in principle be owing to the same process, namely the out-of-Africa expansion [10]. Atkinson also proposed that the spatial origin of human language could be located at the place yielding the best fit between phonemic diversity and distance, and he assumed a linear relationship between both variables. In this way, he located the origin of human language in Africa.

A major motivation of this paper is that previous work on this problem has been based on analysing observed data, but no numerical simulations exist for a *phonemic* serial founder effect model (in contrast to simulations of a *genetic* serial founder effect model [8,9]). However, similar to genetic simulations, linguistic simulations could also be helpful in order to conclude whether a phonemic serial founder effect might have generated the observed spatial trends or not. We report such simulations in this paper, with the hope that they can be useful as a starting point to refuse or accept the viability of a linguistic serial founder effect explanation of the observed non-uniform distribution of phonemic diversity. Numerical simulations can help to establish the conditions under which a serial founder effect might lead to the observed cline (indeed, we shall show that three strong assumptions seem to be necessary). We shall also see that genetic simulations are very different from phonemic ones, so they may lead to different results.

Finally, it is worth noting that Atkinson [5] noted that some theoretical models on linguistic transmission predict that small populations should carry fewer phonemes, either owing to copying errors [11], Darwinian competition [12] or reduced contrastive possibilities [13]. In the following, we show that a global cline of phonemic diversity can arise without the need for any such models. Thus, in our opinion, this paper can be useful also because it exemplifies how numerical simulations can help to distinguish the likely importance of different possible mechanisms at work.

In this paper, we simulate the human dispersal out of Africa about 70 kyr ago [7]. Our idea is very simple. If the hypothesis of a serial founder effect were valid, using reasonable assumptions, anthropologically realistic parameter values for growth and dispersal, an archaeologically and genetically realistic value for the time spent from the onset of the out-of-Africa range expansion, and an empirical value for the phonemic accumulation rate (see the parameter values below), we should be able to obtain a worldwide distribution of phonemic diversity similar to the observed one. On the contrary, if such an approach leads to a distribution different from the observed one, then we will conclude that a simple serial founder model cannot explain the observed pattern.

We shall find that a simple model generates a phonemic cline. Intuitively, our model works as follows. First, we follow other authors in assuming that the number of phonemes increases in time. However, we also assume that this does not happen for all languages, but only for those in regions where the population density is high enough (several previous empirical and theoretical results support this second assumption). In contrast, in regions where some populations have arrived, but the population density is still low, phonemes are not accumulated. In other words, languages on the population front tend to accumulate few phonemes, and populations speaking these low-diversity languages diffuse and propagate the front further away. This is in sharp contrast to regions near the origin of the out-of-Africa expansion (populations there lie behind the front, and are therefore less affected by this process). In this way, we shall see that a rather simple model (explained below in detail) generates a cline of a decreasing number of phonemes per language with increasing distance from the origin of the dispersal of modern humans, and that this cline is consistent with the observed one. Besides discussing *qualitative* features of this and other models (which can be understood intuitively), we shall also perform simulations, so that we can make *quantitative* comparisons (between the cline resulting from each model and the observed one). If a model yields a cline similar to the observed one, such quantitative comparisons yield a precise assessment of its validity.

## 2. Methods

### 2.1. The observed phonemic cline

**Figure 1.**
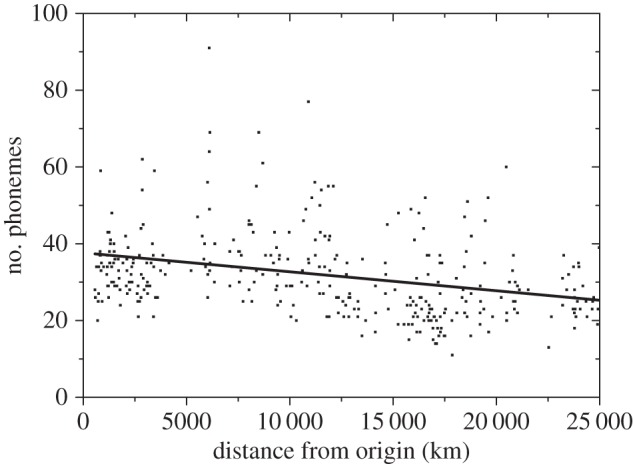
Plot of the number of phonemes of 366 present languages versus their distances from the most likely origin of the out-of-Africa dispersal. Phonemic data have been obtained from the UPSID. The linear fit (straight line) has slope = −(3.4–6.5)×10^−4^ phonemes per km, intercept = 35.4–39.9 phonemes (95% confidence-level intervals), *r* = −0.313, *n* = 366. The slope is very highly significantly different from zero (*p* < 0.001). The database is available as electronic supplementary material, table S1. Other databases yield similar results (electronic supplementary material, figures S1 and S2).

The analysis owing to Atkinson [5] relies on data drawn from the WALS database [14]. However, WALS data do not provide a detailed description of the number of phonemes of each language. For example, for vowels, it only reports if each language has low, average or large vowel diversity. In contrast, the UPSID [15] provides an estimation of the number of phonemes (segments) for each language. We identified 366 languages from the original 504 languages provided by Atkinson [5], whose number of segments were also recorded in the UPSID (we include the database as electronic supplementary material, S1). First, we checked that the feature that we want to model (i.e. the presence of a global cline in phonemic diversity) was still present in the reduced dataset. In figure 1, the number of phonemes of each language is plotted as a function of its average distance from the putative African origin proposed by Atkinson [5]. Those distances are not all measured along great circles, but some of them pass through five waypoints, in order to take into account the role of oceans on plausible migration routes [5]. The negative correlation between the number of phonemes and distance is statistically significant (*r* = −0.313, *p* < 0.001), albeit somehow weaker than using full dataset and the WALS diversity measure [5]. The linear fit (straight line) has slope = −(3.4–6.5) × 10^−4^ phonemes km^−1^, intercept = 35.4–39.9 phonemes (95% confidence-level intervals). Other databases yield similar linear fits to that in figure 1 (see electronic supplementary material, figures S1 and S2).

### 2.2. The simulated phonemic cline

In order to simulate a serial founder effect, we first estimate the necessary parameter values. The timing of the modern human dispersal out of Africa has long been disputed among scholars, with competing theories about how and when it occurred [16]. We assume that the last out-of-Africa dispersal started about 70 kyr ago [7]. This date is debated [17], but our conclusions do not change assuming other realistic dates (electronic supplementary material, §S3c).

As explained in Introduction, Perreault & Mathew [4] reached quantitative estimates for the rate of accumulation of phonemes. We use the upper (0.38 phonemes per kyr) and lower (0.26 phonemes per kyr) bounds estimated from their linear model [4]. A linear accumulation model means that the process of increase of phonemes is not accelerated in time. In the absence of detailed time trajectories of phonemic inventories, it seems reasonable to begin with this simple model in order to explore the consequences of a serial founder effect on the space–time dynamics of linguistic diversity.

Because we want to focus on the effect of a serial founder effect, for simplicity, we do not include oceans, mountains, nor any other geographical features in the simulations. This was also done in genetic serial founder effect simulations [8,9], which were performed in one-dimensional space. Here, we use two dimensions instead. We have represented the surface of the Earth by a square grid of 1000 × 1000 nodes. The distance between any two first neighbours corresponds to *d* *=* 50 km, because this is the characteristic dispersal distance per generation for pre-industrial populations [18]. Initially, there is population only at the central node. Thus, at the end of the range expansion, the maximum distance reached by the population from the origin along the horizontal (or vertical) direction is 500·50 = 25 000 km (it is of course longer along the diagonal direction, but we shall need only the results along the horizontal direction to compare the model with the observations). This distance of 25 000 km is similar to the maximum distances from Africa in the dataset used (electronic supplementary material, S1).

Hassan [19] collected observations on population densities of hunter–gatherers. They vary widely, so we use an intermediate value of 0.8 people km^−2^ (this is representative of values reported for populations in various continents, such as the Ituri pygmies in Africa, some islanders in Asia, and Californian hunter–gatherers). Because the squared cell centred at each node of our grid has 50·50 = 2500 km^2^, this population density leads to about 2000 people per node. A tribe is usually defined as a reproductive (i.e. highly endogamous) group with a common language, possibly spoken also by other tribes [20]. If each tribe has about 400 people [21], then this implies that each node can have up to *n* = 5 tribes. Each person speaks only one language. All people of each tribe speak the same language. Some tribes may speak the same language, in agreement with ethnographic observations [20]. In the electronic supplementary material (§S3b), we repeat the simulations for a larger value of *n* (namely *n* = 10 tribes per cell 50 × 50 km) and find similar results to those reported below (such a larger value of *n* corresponds to other ethnographically realistic values for the population density and number of individuals per tribe, see electronic supplementary material, §S3*b*). The language of each tribe is represented by a binary string of 60 digits equal to ‘0’ (absence of a particular phoneme) or ‘1’ (presence of that phoneme).

In our simulations, each time step *T* corresponds to one generation, defined as the *mean* age difference between a parent and her/his children, i.e. *T* = 32 year for pre-industrial populations [22]. It has been previously shown that when modelling the spread of population waves of advance, the mean parent–children age difference should be used (rather than the difference for the eldest child) [22]. For each time step and at each occupied node, the following computations are performed, similarly to previous simulations of space–time cultural diversity [23].

#### 2.2.1. Dispersal

For practical reasons (simplicity and computing time), isotropic single-distance dispersal was applied, as follows [18,23]. A randomly selected number of tribes, equal to the nearest integer to the product of the initial number times (1 − *p_e_*)/4, disperses into each of the four neighbouring nodes. The rest of the population stays at the original node. We used the mean value *p_e_* = 0.38 for the persistence (fraction of the population that does not move), as measured for pre-industrial populations [18]. We use tribes (i.e. groups of people speaking the same language) as the unit that disperses also for practical reasons: if different individuals of a tribe dispersed into different cells, then we would have many more languages per cell, and the necessary memory space would exceed the capacity of our computers (moreover, each simulation run would take much longer than 24 h, which is the average duration of a simulation run with our model).

#### 2.2.2. Reproduction

We use a net reproductive rate (births minus deaths) *a* = 1% per year = 0.01 year^−1^, which has been estimated from archaeological data [24] (in electronic supplementary material, §S3e, we apply another reasonable value [25] for *a*, and find that the conclusions do not change). Because low population densities *p*(*x, y, t*) increase exponentially, *p*(*x*, *y*, *t* + *T*) = *e^aT^p*(*x*, *y*, *t*) with *e^aT^* ≈ 1.4 from the values of *a* and *T* above. We implement net reproduction simply by generating new additional tribes such that the final number of tribes at the node considered is the nearest integer to 1.4 times the initial number (thus, using the notation in the ecological literature and our previous work [18], *R*_0_ = 1.4). If this yields a result above the carrying capacity (five tribes per node, see above), then the final number is set equal to the carrying capacity.

#### 2.2.3. Vertical transmission

The string of values ‘0’ or ‘1’ (indicating the absence or presence of a phoneme) from a randomly selected old tribe are all passed to a randomly selected new tribe in the same node. Thus, each new population (tribe) is a clone of its parent population, as in previous non-linguistic simulations [23,24]. Note that this step (vertical transmission) corresponds to the transmission of a given language, not to the formation of a new one.

As explained in more detail at the end of this section, new languages are formed by the phonemic accumulation process, by changing a value ‘0’ (absence of a phoneme) into ‘1’ (presence of that phoneme). Two subsequent accumulation steps are separated by a longer time interval (e.g. every 82 generations, see below) than the steps in §§2.2.1 to 2.2.3 above, which take place once per generation.

Note that in our simple model, speaker community size is fixed for each tribe (400 people), but several tribes may speak the same language (and tribes speaking the same language may be located either at the same spatial cell or at different ones).

This sequence of three processes (dispersal, reproduction and vertical transmission) is repeated, e.g. 2280 times, corresponding to 2280 generations × 32 year generation^−1^ = 72 960 year or about 70 kyr, in agreement with some archaeological estimates of the time elapsed since the onset of the out-of-Africa dispersal until today [7] (other reasonable values yield similar results, see electronic supplementary material, §S3c). When the population reaches a node located at the edge of the grid, the simulation continues simply taking into account that this node cannot exchange individuals with four neighbours but only with three of them (or two of them, in the case of the four nodes located at the corners of the grid).

Languages in the island of Timor (north of Australia) have distances (from Atkinson's best-fit origin) about 15 000 km (electronic supplementary material, S1). Using the values of *d, T, p_e_* and *R*_0_ above, it takes the wave of advance of modern humans (arising from our numerical simulations) about 28 kyr to reach this distance, i.e. it arrives there 42 kyr ago, which is consistent with the dates of archaeological sites in Timor (42–44 kyr BP) [26].

In the electronic supplementary material (§S3), we perform a sensitivity analysis by repeating the simulations for other empirical values of the parameters: besides the population density and tribe population size (which determine the maximum number of tribes per node), we also vary the time elapsed since the out-of-Africa dispersal, the generation time, the initial growth rate, the mobility behaviour, etc. Our main conclusions do not change.

Obviously, it is impossible to perform numerical simulations without setting some languages at the start of the simulation (i.e. at the onset of the out-of-Africa dispersal). They are unfortunately unknown, but in the work by Perreault & Mathew [4] languages with many phonemes are seen as the result of an evolutionary process of phoneme accumulation. Thus, in this framework, it is reasonable to assume that the original language(s) spoken by the modern humans who started the out-of-Africa dispersal had few phonemes. In the absence of any prehistoric linguistic data, we therefore assume that the number of phonemes of those original languages where similar to those of the present languages with the smallest phoneme inventories (as already assumed by Perreault & Mathew [4]). Today, Piraha is the language that has the smallest number of phonemes (11 phonemes) and it could be an archaic example of an early stage in human language evolution [27]. Therefore, 11 phonemes is a reasonable minimum for the phonemic inventory size [4]. As mentioned earlier, at the onset of the simulations, all nodes but the central one were empty of people. The central node was initially populated with five tribes, with 11, 11, 13, 13 and 14 phonemes, respectively, which are the observed numbers of phonemes for the five present languages with the lowest number of phonemes [15] (electronic supplementary material, table S1). In electronic supplementary material, §S3a we repeat the simulations assuming that all initial tribes spoke the lowest-diversity language (11 phonemes) and find similar results.

**Figure 2.**
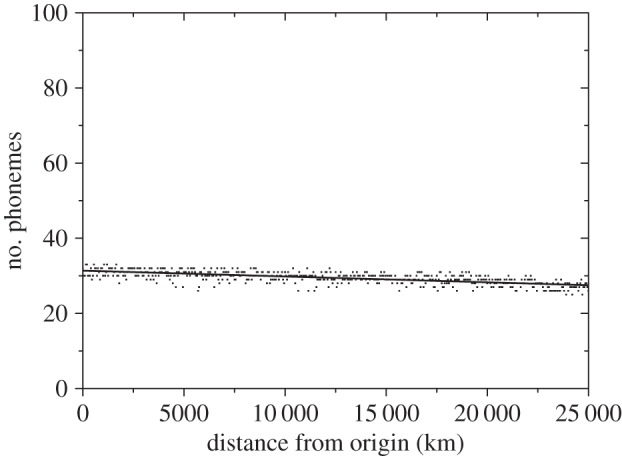
Simulated number of phonemes at present (*t* = 2280 generations ≈ 70 kyr) versus distance from the most likely origin of the out-of-Africa dispersal, for the observed lower bound of the rate of phonemic accumulation (0.26 phonemes per kyr, i.e. one phoneme added each 120 generations). The linear fit (straight line) has slope = −(1.4–1.7) × 10^−4^ phonemes per km, intercept = 31.2–31.6 phonemes (95% confidence-level intervals), *r* = −0.671, *n* = 501, *p* < 0.001.

**Figure 3.**
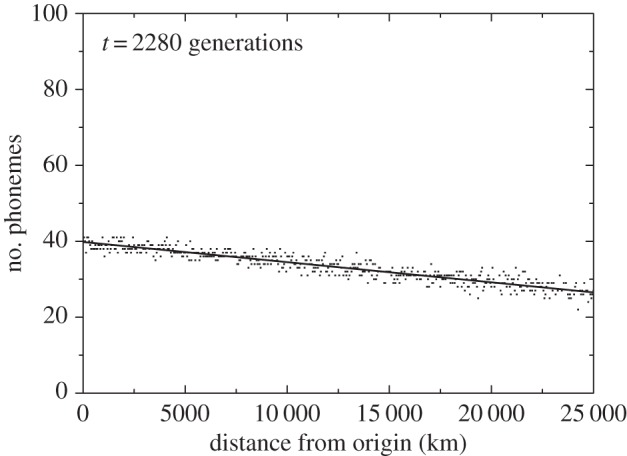
Simulated number of phonemes at present (*t* = 2280 generations ≈ 70 kyr) versus distance from the most likely origin of the out-of-Africa dispersal, for the observed upper bound of the rate of phonemic accumulation (0.38 phonemes per kyr, i.e. one phoneme added each 82 generations). The linear fit (straight line) has slope = −(5.1–5.5) × 10^−4^ phonemes km^−1^, intercept = 39.5–40.1 phonemes (95% confidence-level intervals), *r* = −0.929, *n* = 501, *p* < 0.001.

We constructed four increasingly complex models, so that the effects owing to different causes can be analysed separately. Only model 3 in the following has been used to obtain the main results (figures 2 and 3).

As mentioned in Introduction, Perreault & Mathew [4] used linguistic and archaeological data from real populations to estimate a rate of increase (i.e. an accumulation rate) for the number of phonemes. Our simplest model (model 1, see electronic supplementary material, §S2a for details) ignores this possibility, i.e. it has a vanishing phonemic accumulation rate. As expected, no cline was generated. However, a long time before the population reached the end of the grid, only a single language was present at sufficiently large distances from the origin of the dispersal along each direction. This spatial drift effect is due to the fact that not all languages from a cell disperse to *all* four nearest neighbouring cells. For example, for the parameter values given above, if a cell has five languages, one language will disperse to each of the four nearest cells (i.e. along the four vertical/horizontal directions), and the fifth one will remain at the original cell. Therefore, as the population wave of advance spreads from the centre to the edges of the grid, this selection (drift) effect takes place many times. We are thus dealing with a repeated bottleneck effect, which explains that a single language is selected at large distance from the origin of the dispersal (this is seen graphically in a series of snapshots displaying the number of phonemes of languages versus distance at several times in electronic supplementary material, figure S3).

In another simple model (model 2, electronic supplementary material, §S2b), all languages accumulated phonemes and did so at the same rate. Again as expected, no cline was generated but a gradual increase in the number of phonemes is obtained (for some snapshots with the number of phonemes versus distance at several times, see electronic supplementary material, figure S4). Let us stress that in our models with phonemic accumulation, we do not attempt to model the particular mechanisms by which languages increase or decrease their number of phonemes, but we do include the net effect of these mechanisms on the phonemic diversity with a single averaged rate of accumulation of phonemes (following Perreault & Mathew [4]).

In our model 3 (electronic supplementary material, §S2c), which has been used to obtain the results reported below, we assumed that only languages with high speaker *densities* increase their number of phonemes. From the beginning of the simulation, the process of accumulation of phonemes takes place in all cells where the population density has already reached carrying capacity, by randomly adding one extra phoneme to each language (i.e. a digit ‘0’ is turned into ‘1’) at the prescribed rate of one phoneme added each 82 generations (higher bound) or 120 generations (lower bound). These bounds correspond, respectively, to the upper (0.38 phonemes per kyr) and lower (0.26 phonemes per kyr) bounds estimated by Perreault & Mathew [4]. The model that generates a global phonemic cline (model 3) is described in more detail in electronic supplementary material, §S2*c* (for some snapshots with the number of phonemes versus distance at several times, see electronic supplementary material, figure S5; for a graphical explanation of the origin of the cline, see electronic supplementary material, figure S6). In electronic supplementary material, §S2c, it is also shown that it is equivalent to consider high population densities or high speaker densities (because we assume that each tribe occupies its own area, without sharing it with any other tribe, so that languages do not overlap geographically).

The results reported here (figures 2 and 3) have been obtained assuming that phonemic accumulation takes place only at cells at saturation density. However, similar results are obtained if we assume a lower accumulation threshold, i.e. a lower value for the speaker density above which languages accumulate phonemes (electronic supplementary material, §S3*g*).

Finally, in our model 4, only languages with large *numbe*rs of speakers (instead of high speaker *densities*) accumulate phonemes. This model did not generate a cline consistent with the observed one, because the number of phonemes increases with distance from the origin of the out-of-Africa expansion (see electronic supplementary material, §S2*d*, especially figure S7), whereas the observed cline has the opposite trend, i.e. the number of phonemes decreases with increasing distance (figure 1). This result of model 4 can be understood as follows. As explained above, spatial drift causes the selection of a single language at large distances (in models 2–4, this happens from the addition of a phoneme until that of the next one, 82 or 120 generations later). Hence, cells *at large distances* will all have the same language. If such a cell has a sufficient number of tribes, then a new phoneme will be added to their language (in model 4). In contrast, in the cells close to the origin of the dispersal (*low distances*), there are several languages, so each of those languages has less speakers. None of those will add a new phoneme, because in each cell, languages spoken by few tribes do not add a phoneme (in model 4). Thus, in model 4, tribes at *low distances* remain with few phonemes, whereas tribes at *large distances* accumulate phonemes. This explains why model 4 generates a cline of increasing phonemic diversity (electronic supplementary material, figure S7), contrary to the observations (figure 1). We stress that we have not used model 4 to obtain the main results (figures 2 and 3). However, model 4 is useful because it allows us to conclude that a cline consistent with the observed one is obtained only if languages with high speaker *densities* (not with large *numbe*rs of speakers) accumulate phonemes.

Admittedly, we have no proof of the validity of our assumption that the process of phonemic accumulation requires high population densities. However, an increase of cultural diversity with population density has been repeatedly invoked to explain many other observations in world prehistory and history, such as the spatial distribution of mode 1 and mode 2 assemblages during the Lower Pleistocene [28], the appearance of modern human behaviour [29], the appearance of social stratification and regional institutions [30], etc. It has been also proposed, on the basis of empirical data, that there is a threshold in the population density of hunter–gatherers above which cultural evolution processes switch on [31]. Cultural transmission models also predict that cultural accumulation increases with population density [32]. Concerning linguistic studies, it has been argued that empirical data show that a higher degree of linguistic contact leads to a higher phonemic diversity, and this has been used to suggest that Atkinson's cline may not be the result of languages gradually losing their phonemes, but rather of languages staying behind the waves of migration gradually gaining phonemes with the increase in population density [33]. Thus, our assumption that phonemic accumulation grows with population density, as well as the possibility that this may explain the global phonemic cline, have been already proposed previously by linguists [33]. This is essentially the idea that our model formalizes quantitatively (for some snapshots of the simulated phonemic cline at different times, where the gradual increase of phonemes can be appreciated, see the electronic supplementary material, figure S5). In any case, we think that our work is only a first step showing the usefulness of simulation approaches to analyse quantitatively the proposal of a serial founder effect of language expansion from Africa [5], in the sense of showing if is possible to generate a cline from clear assumptions, as well as to compare it to the observed cline. To the best of our knowledge, none of both quantitative analyses has been performed previously.

## 3. Results and discussion

Figures 2 and 3 show the results of the simulations, i.e. the number of phonemes of each language as a function of its distance from the origin after 70 kyr [7] of the onset of the out-of-Africa dispersal. For the sake of clarity, we plot only one language per node along the horizontal direction (i.e. a total of 501 simulated languages). Figure 2 has been obtained by setting the rate of accumulation of phonemes to the lower observed bound (0.26 phonemes per kyr, i.e. one phoneme is added each 120 generations). Similarly, figure 3 has been obtained for the upper bound of the phoneme accumulation rate (0.38 phonemes per kyr, i.e. one phoneme is added each 82 generations). Note that all five initial languages (with 11, 11, 13, 13 and 14 phonemes) are eventually lost, because all of the final languages have more than 20 phonemes (figures 2 and 3). This is due to the process of phonemic accumulation.

The plot obtained from the languages spoken today worldwide (figure 1) and the two plots obtained from our simulations (figures 2 and 3) all display a cline of decreasing phonemic diversity with increasing distance from the presumed origin of the out-of-Africa expansion. The slopes of all three clines are very highly significantly different from zero (*p* < 0.001).

The intercept of the linear fit in figure 2 is lower than in figure 3, as expected, because fewer phonemes are added per unit time in figure 2 than in figure 3. The intercept according to the observed data (caption to figure 1) indicates that, on average, present African languages have about 35.4–39.9 phonemes (95% confidence-level interval). This range is consistent with the 39.5–40.1 phonemes predicted by the intercept of the linear fit to the cline simulated using the upper bound of the observed accumulation rate (figure 3). We note, however, that the lower bound of the observed accumulation rate (figure 2) yields an intercept (31.2–31.6 phonemes) that is too small to be consistent with the observations (35.4–39.9 phonemes, from figure 1).

We can understand intuitively the values of the intercepts in figures 2 and 3 as follows. The initial languages used in our simulations have 11, 11, 13, 13 and 14 phonemes (§2). Figure 2 has been obtained by adding 0.26 phonemes kyr^−1^, which implies about 18 phonemes after 70 kyr. If we add up this number to the average initial inventories (11–14 phonemes), then we obtain 29–32 phonemes, which is consistent with the intercept from the simulations, i.e. 31.2–31.6 phonemes (figure 2). Similarly, figure 3 has been obtained by adding 0.38 phonemes kyr^−1^, which implies about 27 phonemes after 70 kyr. Adding up the average initial inventories (11–14 phonemes), we obtain 38–41 phonemes, which is consistent with the intercept from the simulations, i.e. 39.5–40.1 phonemes (caption to figure 3). Therefore, phonemic accumulation explains the intercept of the cline, whereas drift (the serial founder effect) has an influence on its slope (as estimated in the next paragraph). The agreement, for an accumulation rate within the observed range (but sufficiently close to its upper bound), of the intercept from the simulations (figure 3) and from the data (figure 1) seems noteworthy, given the fact that the phonemic accumulation rates used in the simulations (0.26–0.38 phonemes kyr^−1^) were not originally estimated [4] by computing the difference between the average number of phonemes of highest-diversity and lowest-diversity languages (and dividing by 70 kyr) but, instead, by comparing the average phonemic inventories in mainland Southeast Asia and the Andaman Islands (and dividing by the time elapsed since their separation, estimated as 45–65 ky) [4], as explained in our Introduction.

The slope of the linear fit to the simulations is −(1.4–1.7) × 10^−4^ phonemes km^−1^ if the simulations are performed using the lower bound of the observed rate of phonemic accumulation (figure 2), and −(5.1–5.5) × 10^−4^ phonemes km^−1^ if using the upper one (figure 3; 95% confidence-level intervals). Again, only the result for the upper (or fastest) bound to the observed accumulation rate is consistent with the slope of the observed global phonemic pattern, namely −(3.4–6.5) × 10^−4^ phonemes km^−1^ (figure 1). We have not used any free or adjustable parameters. Instead, all parameter values in the simulations have been estimated from independent observations (§2). From this perspective, the agreement between the slopes of the simulated cline (for a sufficiently fast accumulation rate, figure 3) and the observed one (figure 1) is encouraging.

Finally, we note that the correlation coefficients are higher for the simulation results (figures 2 and 3) than for the observed data (figure 1), because the observed languages lie, on average, further away from the regression line (figure 1) than the simulated ones (figures 2 and 3). This is not surprising, because the simulations have been performed on a regular grid, thus oceans and seas are not taken into account in the simulations (figures 2 and 3). Also, the simulations assume isotropic and homogeneous dispersal (so barriers such as mountains and deserts are not included). Similarly, the possibility of fastest migration routes along coasts and rivers [34–36] is not taken into account in the simulations. Also, for simplicity, in this paper, we have assumed that all populations that migrate move the same distance, so the effect of more detailed dispersal distributions [37] is not included in the simulation results. All such factors, as well as possibly non-homogeneous and non-steady rates of phonemic change, the demographic transition into agriculture, linguistic range expansions and other prehistoric and historic processes, could have affected the observed pattern (figure 1). Thus, it is not surprising that the simulations (figures 2 and 3) have higher correlations than the data (figure 1). In spite of its limitations, our simple model is able to predict a phonemic diversity cline similar to the observed one (figure 1), but only for a sufficiently fast accumulation rate (figure 3).

These results clarify two objections that have been raised against Atkinson's proposal of a phonemic serial founder effect [5]. First, as noted by Pericliev [38], ‘one wonders why the number of phonemes must diminish in a founder population (…) A group of people will quite probably carry the WHOLE phonemic inventory to the new place of residence, and not only part of this inventory’ (see also pp. 150–151 in the criticism by Bybee [39]). This crucial point is very reasonable and is not a problem in our simulations, where migrating people do indeed carry the whole phonemic inventory to the new place of residence.

Our simulations also clarify a related point. In order to justify the phonemic serial effect, it was originally proposed that phonemes are most likely to be lost in small founder populations [5,40]. However, this is not necessary because it is not assumed in our simulations (see also §4).

A second criticism [39] to the possibility of a serial founder effect in phonemics (in contrast to genetics) is that the same language is shared by all individuals of a population, whereas genes are not (i.e. each individual has his/her own genes, but all individuals have the same language). This difference is taken into account in our simulations because we deal with tribes, and all individuals in a tribe speak the same language. Thus, the criticism based on this difference between phonemes and genes [39] is also reasonable and may apply to some models, but not to the simulations reported here.

Let us stress that, in order to estimate a range for the accumulation rate of phonemes, Perreault & Mathew [4] had to assume that the process of phonemic accumulation did not take place in the Andaman Islands. As explained in Introduction, this could have happened owing to one or several factors that made language evolution in the Andaman Islands different from Mainland Southeast Asia, e.g. the population size [4], the mean number of speakers per language, the population density, the social network structure (which is possibly related to geography), etc. Of course, if we suppressed the process of phonemic accumulation in some regions of our lattice (without need to assume any specific reason), the simulations would yield less phonemes today in those regions, when compared with others at similar distances from Africa. Similarly, if our simulations were performed in a real geography (instead of a square lattice of cells) and we assumed that phonemic accumulation did not take place in the Andaman Islands (without need to assume any specific reason), then we would obtain substantially less phonemes in the Andaman Islands when compared with Mainland Southeast Asia (in agreement with the data observed by Perreault & Mathew [4]). It is true that, alternatively, we could assume that the population density in the Andaman Islands remained below saturation for most of the 45–65 kyr since the arrival of modern humans. In this case, our model would explain why the Andaman Islands have lower phonemic diversity today than Mainland Southeast Asia. However, we have no data on the population density in the Andaman Islands during the 45–65 kyr since the arrival of modern humans. Therefore, we do not claim that the population density was low in the Andaman Islands and that this suppressed phonemic accumulation. We prefer to admit that there are other possible causes. Our results are only a first step showing only that phonemic accumulation may be linked to the serial founder effect (and that this may lead to the observed global phonemic cline), but we do not attempt to explain the current phonemic diversity in specific regions (e.g. the Andaman Islands) on which we lack sufficiently detailed information.

## 4. Conclusion

The simulations reported in this paper are, to the best of our knowledge, the first ones of a *phonemic* serial founder effect model out of Africa. They show that such an effect could, in principle, have generated the observed phonemic diversity cline. However, we must caution that this result has been found under three strong assumptions: (i) a phonemic accumulation rate close to the upper bound estimated by Perreault and Mathew from phonemic and archaeological data [4]; (ii) that languages at the onset of the out-of-Africa dispersal had low phonemic diversities (as also assumed by Perreault & Mathew [4]) and (iii) that the phonemic accumulation rate is faster for languages with high numbers of speakers per unit area (as suggested by Coupé *et al.* [33]). These three assumptions are possible, in principle, but not well established. Thus, obviously, we cannot claim that a phonemic serial founder effect out of Africa generated the observed cline. Our work should be regarded as only a starting point, with the hope that alternative models (with or without a serial founder effect) will be analysed in the future via numerical simulations. This should clarify whether only the above-mentioned three assumptions can reproduce the observed cline, or otherwise which alternative models can explain it (and, if so, under which assumptions).

We have not assumed any of the existing theoretical linguistic transmission models in which small populations lose more phonemes, e.g. owing to copying errors [11], Darwinian competition [12] or reduced contrastive possibilities [13]. We would like to stress, however, that numerical simulations should be performed in order to see if any of those models could yield a cline similar to the observed one or not, and under which assumptions. Clearly, many additional simulations will be necessary to compare different models and assumptions. A crucial difference to the present paper is that, in those alternative models [11–13], phonemes are lost by languages (specially those with small populations of speakers). In contrast, in our simulations, we do not assume that languages lose any phonemes (neither that phonemes are lost if a small subset of a population separates from the rest). In our simulations, languages have less phonemes at further distances (from the origin of the population dispersal), simply because languages propagating on the front (i.e. languages of pioneering, low population–density populations) accumulate less phonemes that languages behind the front (i.e. languages in regions with population density equal to its maximum possible value) (see electronic supplementary material, figure S6 for a graphical explanation).

It has been noted by Sproat [41] that a phonemic cline can arise from a serial founder effect either because small populations lose diversity or because large populations gain it. The former possibility corresponds to Atkinson's original proposal [5,40]. The second one corresponds to this paper (and was suggested previously by Coupé *et al.* [33]). It remains to be seen if numerical simulations based on the first proposal can also generate a cline similar to the observed one, and under which assumptions.

Note that our *phonemic* simulations are rather different from *genetic* simulations of a serial founder effect [8,9]. We are dealing with phonemes and, for this reason, we have applied the phonemic accumulation rate estimated by Perreault & Mathew [4]. This is not analogous to applying a mutation rate to genes, because in our case, a new phoneme is *added* in a given time interval. In contrast, in the genetic case, an allele is *replaced* by another one (e.g. by increasing or decreasing the number of repeats in one of 783 simulated microsatellite genes in each individual [8,9]). Moreover, in our case, the addition of a phoneme applies to the language spoken by *all* individuals of a tribe, whereas in the genetic case, the mutation affects only *a single* newborn individual. Thus, the results from genetic simulations cannot be extrapolated to phonemic dynamics.

The above-described simulations could be generalized to real geographies in order to analyse the effects of landmasses (oceans, mountains, etc.) on the simulated cline (figures 2 and 3), as well as on patterns of phonemic diversity within regions (e.g. for the Austronesian languages [1]). They could also be extended to test different hypothetical dispersal scenarios (migration routes), as well as the geographical variability of founder effects and of phonemic accumulation rates.

It has been claimed by some researchers [39,41–44] that rates of phonemic change are so high that any founder-effect signal would quickly disappear after the out-of-Africa expansion. Their position is reasonable on the basis of specific examples showing that the number of phonemes can change quickly, e.g. from proto-Indo-European into the Indo-European languages [43], from proto-Bantu into the Bantu languages [41], or even from Latin into the Romance languages [39]. However, we would like to point out that (i) the observed cline (figure 1) calls for an explanation; (ii) it refers to the average diversity at each distance from Africa (not to an exact relationship for all languages) and (iii) those authors [39,41–44] did not apparently perform any spatial simulation to support their view quantitatively. In our spatial simulations, we naturally find a phonemic diversity cline 70 kyr after the onset of the out-of-Africa expansion (figure 3) similar to the observed one (figure 1), thus the founder-effect signal does not disappear quickly enough to become undetectable at present (provided that the accumulation rate is fast enough). But, our model is admittedly very simple (as necessary to understand the consequences of a serial founder effect). We do not include processes such as, e.g. the evolution from proto-Indo-European into the Indo-European languages. Is it possible to generalize our simulations to include such processes? How would the simulated phonemic cline (and thus the conclusions) change? Would this simply increase the scatter, i.e. the value of *r* in the captions to figures 2 and 3 (thus becoming closer to that in figure 1)? Or would the simulated cline disappear completely (leading to much higher values of *P* in the captions to figures 2 and 3), as expected by some authors [39,41–44]? The results of such more complicated simulations could perhaps be compared also with statistical properties of the spatial distribution of languages and language families, as well as to additional properties of languages (e.g. grammar). These are interesting problems that deserve future work.

Further simulations will hopefully tackle many other interesting questions. We mention a few of them. Does a simulation out of Africa always yield a higher correlation coefficient and/or higher AIC/BIC support in Africa? (Such criteria have been applied by some authors [3,5,42].) If it does, will realistic later population movements and/or language substitutions change this conclusion? How long does it take for the phonemic cline to disappear? How does this timescale compare with the corresponding one for the *genetic* worldwide cline [8]? What other implications follow from the differences between linguistic and genetic evolution, in terms of the formation, shape and dynamics of their respective clines? Is the recently reported [3] lack of correlation above 10 000 km necessarily inconsistent with a serial founder effect, as recently suggested [45]? Can spatial numerical simulations be used to analyse the predictions of proposed alternative explanations of the phonemic cline [40,43,44,46,47], compute the cline generated by each proposal, and compare that simulated cline with the observed one? (As done in this paper for a specific serial founder effect proposal.) Numerical simulations can obviously help to answer these (and many other) interesting questions. Clearly, this opens a wide field of spatial phonemic simulation that can be used to analyse and clarify the implications of the out-of-Africa dispersal (and subsequent processes) on patterns of phonemic diversity.

Before closing, we would like to stress the following point. It is true that qualitative features of different models can be understood intuitively. However, numerical simulations (such as those reported in this paper and its electronic supplementary material) are absolutely necessary to compare quantitatively the cline obtained from each model to the observed one (here we have performed such a comparison using the slope, intercept and values of *r* and *P* of linear fits, figures 1–3). If a model yields a cline similar to the observed one, then quantitative comparisons yield a precise assessment of its validity. In our opinion, quantitative comparisons (of this or other kinds) are of utmost importance to assess the usefulness (or uselessness) of different models to explain the worldwide phonemic cline.

## Supplementary Material

Database

## Supplementary Material

Supplementary text, tables and figures

## References

[1] Trudgill P 2004 Linguistic and social typology: the Austronesian migrations and phoneme inventories. Linguist. Typol. 8, 305–320. (10.1515/lity.2004.8.3.305)

[2] Hay J, Bauer L 2007 Phoneme inventory size and population size. Language 83, 388–400. (10.1353/lan.2007.0071)

[3] Creanza N, Ruhlen M, Pemberton TJ, Rosenberg NA, Feldman MW, Ramachandran S 2015 A comparison of worldwide phonemic and genetic variation in human populations. Proc. Natl Acad. Sci. USA 112, 1265–1272. (10.1073/pnas.1424033112) 25605893 PMC4321277

[4] Perreault C, Mathew S 2012 Dating the origin of language using phonemic diversity. PLoS ONE 7, e35289 (10.1371/journal.pone.0035289) 22558135 PMC3338724

[5] Atkinson QD 2001 Phonemic diversity supports a serial founder effect model of language expansion from Africa. Science 332, 346–349. (10.1126/science.1199295) 21493858

[6] Stewart JR, Stringer CB 2012 Human evolution out of Africa: the role of refugia and climate change. Science 335, 1317–1321. (10.1126/science.1215627) 22422974

[7] Oppenheimer S 2012 Out-of-Africa, the peopling of continents and islands: tracing uniparental gene trees across the map. Phil. Trans. R. Soc. B 367, 770–784. (10.1098/rstb.2011.0306) 22312044 PMC3267120

[8] Ramachandran S, Deshpande O, Roseman CC, Rosenberg NA, Feldman MW, Cavalli-Sforza LL 2005 Support from the relationship of genetic and geographic distance in human populations for a serial founder effect originating in Africa. Proc. Natl Acad. Sci. USA 102, 15 942–15 947. (10.1073/pnas.0507611102) 16243969 PMC1276087

[9] Deshpande O, Batzoglou S, Feldman MW, Cavalli-Sforza LL 2009 A serial founder effect model for human settlement out of Africa. Proc. R. Soc. B 276, 291–300. (10.1098/rspb.2008.0750) PMC2674957 18796400

[10] Henn BM, Cavalli-Sforza LL, Feldman MW 2012 The great human expansion. Proc. Natl Acad. Sci. USA 109, 17 758–17 764. (10.1073/pnas.1212380109) PMC3497766 23077256

[11] De Boer B 2000 Self-organization in vowel systems. J. Phonet. 28, 441 (10.1006/jpho.2000.0125)

[12] Ritt N 2004 Selfish sounds and linguistic evolution: a Darwinian approach to language change. New York, NY: Cambridge University Press.

[13] Trudgill P 2011 Social structure and phoneme inventories. Linguist. Typol. 15, 155–160. (10.1515/lity.2011.010)

[14] Dryer MS, Martin H. (eds). 2013 The world atlas of language structures online. Leipzig: Max Planck Institute for Evolutionary Anthropology See http://wals.info.

[15] Maddieson I, Precoda K 1990 Updating UPSID. UCLA working papers in Phonetics 74, 104–111. We obtained the phoneme inventory data from the website by prof. Henning Reetz. See http://web.phonetik.uni-frankfurt.de/upsid_nr_seg.html.

[16] Reyes-Centeno H, Ghirotto S, Détroit F, Grimaud-Hervé D, Barbujani D, Harvati K 2014 Genomic and cranial phenotype data support multiple modern human dispersals from Africa and a southern route into Asia. Proc. Natl Acad. Sci. USA 111, 7248–7253. (10.1073/pnas.1323666111) 24753576 PMC4034217

[17] Mellars P, Gori KC, Carr M, Soares PA, Ricards MB 2013 Genetic and archaeological perspectives on the initial modern human colonization of southern Asia. Proc. Natl Acad. Sci. USA 110, 10 699–10 704. (10.1073/pnas.1306043110) PMC3696785 23754394

[18] Fort J, Pérez-Losada J, Isern N 2007 Front from integro-differential equations and persistence effects on the Neolithic transition. Phys. Rev. E 76, 031913 (10.1103/PhysRevE.76.031913) 17930277

[19] Hassan FA 1981 Demographic archaeology. New York, NY: Academic Press.

[20] Dixon RMW 1976 Tribes, languages and other boundaries in northeast Queensland. In Tribes and boundaries in Australia (ed. Nicolas P), pp. 207–238. Canberra, Australia: Australian institute of aboriginal studies.

[21] Lee RB, deVore I. (eds). 1968 Man the hunter. Chicago, IL: Aldine.

[22] Fort J, Jana D, Humet J 2004a Multi-delayed random walks: theory and application in Neolithic transition in Europe. Phys. Rev. E 70, 031913 (10.1103/PhysRevE.70.031913) 15524555

[23] Pérez-Losada J, Fort J 2011 Spatial dimensions increase the effect of cultural drift. J. Arch. Sci. 38, 1294–1299. (10.1016/j.jas.2011.01.004)

[24] Conolly J, Colledge S, Shennan S 2008 Founder effect, drift, and adaptive change in domestic crop use in early Neolithic Europe. J. Arch. Sci. 35, 2797–2804. (10.1016/j.jas.2008.05.006)

[25] Fort J, Pujol T, Cavalli-Sforza LL 2004 Palaeolithic populations waves of advance. Camb. Archaeol. J. 14, 53–61. (10.1017/S0959774304000046)

[26] O'Connell JF, Allen J 2012 The restaurant at the end of the universe: modelling the colonisation of Sahul. Australian Archaeol. 74, 5–17.

[27] Everett D 2005 Cultural constraints on grammar and cognition in Pirahã: another look at the design features of human language. Curr. Anthropol. 46, 621–646. (10.1086/431525)

[28] Romanowska I, Gamble C, Bullock S, Sturt F 2016 Dispersal and the Movius line: testing the effect of dispersal on population density through simulation. Quatern. Int. (10.1016/j.quaint.2016.01.016)

[29] Powell A, Shennan S, Thomas JG 2009 Late Pleistocene demography and the appearance of modern human behavior. Science 324, 1298–1301. (10.1126/science.1170165) 19498164

[30] Johnson AW, Earle TK 1987 The evolution of human societies: from foraging group to agrarian state. Stanford, CA: Stanford University Press.

[31] Collard M, Ruttle A, Buchanan B, O'Brien MJ 2013 Population size and cultural evolution in nonindustrial food-producing societies. PLoS ONE 8, e72628 (10.1371/journal.pone.0072628) 24069153 PMC3772076

[32] Powell A, Shennan S, Thomas JG 2010 Demography and variation in the accumulation of culturally inherited skills. In Innovation in cultural systems: contributions from evolutionary anthropology (eds O'Brien MJ, Shennan S), pp. 139–160. Cambridge, MA: MIT Press.

[33] Coupé C, Hombert J-M, Marisco E, Pellegrino F 2013 Investigations into determinants of the diversity of the world's languages. In Eastward flows the great river (eds Gang P, Feng S), pp. 75–108. Hong Kong: City University of Hong Kong Press.

[34] Zilhao J 2001 Radiocarbon evidence for maritime pioneer colonization at the origins of farming in west Mediterranean Europe. Proc. Natl Acad. Sci. USA 98, 14 180–14 185. (10.1073/pnas.241522898) 11707599 PMC61188

[35] Fort J, Pujol T, Vander Linden M 2012 Modelling the Neolithic transition in the Near East and Europe. Am. Antiq. 77, 203–220. (10.7183/0002-7316.77.2.203)

[36] Davison K, Dolukhanov P, Sarson GR, Shukurov A 2006 The role of waterways in the spread of the Neolithic. J. Archaeol. Sci. 33, 3641 (10.1016/j.jas.2005.09.017)

[37] Fort J 2012 Synthesis between demic and cultural diffusion in the Neolithic transition in Europe. Proc. Natl Acad. Sci. USA 109, 18 669–18 673. (10.1073/pnas.1200662109) 23112147 PMC3503213

[38] Pericliev V 2011 On phonemic diversity and the origin of language in Africa. Linguist. Typol. 15, 217–222. (10.1515/lity.2011.016)

[39] Bybee J 2011 How plausible is the hypothesis that population size and dispersal are related to phoneme inventory size? Linguist. Typol. 15, 147–154. (10.1515/lity.2011.009)

[40] Atkinson QD 2011 Linking spatial patterns of language variation to ancient demography and population migrations. Linguist. Typol. 15, 321–332. (10.1515/lity.2011.022)

[41] Sproat R 2011 Phonemic diversity and the out-of-Africa theory. Linguist. Typol. 15, 199–206. (10.1515/lity.2011.014)

[42] Hunley K, Bowern C, Healy M 2012 Rejection of a serial founder effects model of genetic and linguistic evolution. Proc. R. Soc. B 279, 2281–2288. (10.1098/rspb.2011.2296) PMC3321699 22298843

[43] Dahl O 2011 Are small languages more or less complex than big ones? Linguist. Typol. 15, 171–175. (10.1515/lity.2011.012)

[44] Bowern C 2011 Out of Africa? The logic of phoneme inventories and founder effects. Linguist. Typol. 15, 207–216. (10.1515/lity.2011.015)

[45] Hunley K 2015 Reassessment of global gene–language coevolution. Proc. Natl Acad. Sci. USA 112, 1919–1920. (10.1073/pnas.1425000112) 25675520 PMC4343119

[46] Wichmann S, Rama T, Holman EW 2011 Phonological diversity, word length, and population sizes across languages: the ASJP evidence. Linguist. Typol. 15, 177–198. (10.1515/lity.2011.013)

[47] Maddieson I, Bhattacharya T, Smith EE, Croft W 2011 Geographical distribution of phonological complexity. Linguist. Typol. 15, 267–280. (10.1515/lity.2011.020)

