# Supplementary material for: Can a linguistic serial founder effect originating in Africa explain the worldwide phonemic cline?
Source: J R Soc Interface. 2016 Apr;13(117):20160185. doi: 10.1098/rsif.2016.0185 (PMC4874439; doi:10.1098/rsif.2016.0185)
Supplement: Supplementary text, tables and figures [file rsif20160185supp2.pdf]

Supplementary information S2

S1. Effect of the database used

In a separate excel file (Supp. Info. S1) we include the database used to prepare Fig. 1 in the main paper. In some cases, the number of phonemes of a given language is not the same in all databases, because of the different criteria applied by their authors. In order to see whether such differences have an effect on the intercept and slope of the observed cline, in Figs. S1-S2 we repeat the linear fit in Fig. 1 but using two other databases (instead of the UPSID database included as Supp. Info. S1). We note from Figs. S1-S2 that the intercept and slope of the fit are not strongly affected by the database used. Thus the conclusions in the main paper remain valid.

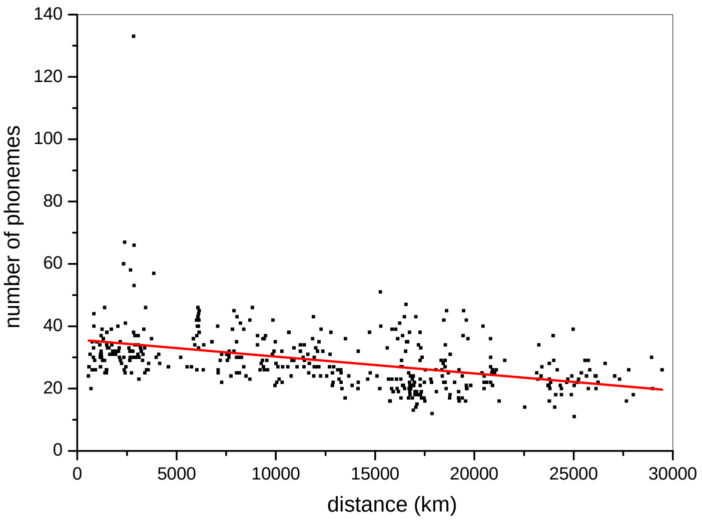

Fig. S1. This figure is the same as Fig. 1 in the main paper, but based on the Ruhlen database[1] (instead of on the database included as Supp. Info. 1 to the present paper). We identified 405 languages from the original 504 languages provided by Atkinson[2], whose number of segments were recorded in the Ruhlen database[1]. For comparison purposes, the distances are those given by Atkinson[2] (as in Fig. 1 in the main paper), i.e. the spatial origin is that suggested by Atkinson[2]. The intercept is 34.0-37.4 phonemes (as compared to 35.4-39.9 phonemes in Fig. 1). The slope is -(4.3-6.6)$\cdot 10^{-4}$ phonemes/km (as compared to -(3.4-6.5)$\cdot 10^{-4}$ phonemes/km in Fig. 1). The correlation

coefficient is -0.43 (as compared to -0.32 in Fig. 1). $P < 0.001$. All intervals have been computed with 95% confidence level.

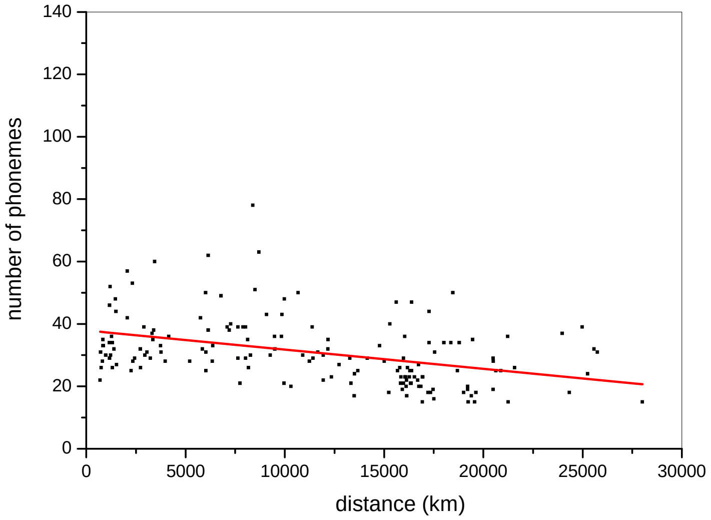

Fig. S2. This figure is the same as Fig. 1 in the main paper, but based on a third database instead[3] (instead of on the database included as Supp. Info. 1 to the present paper). We identified 159 languages from the original 504 languages provided by Atkinson[2], whose number of segments were recorded in the third database[3]. For comparison purposes, the distances are those given by Atkinson[2] (as in Fig. 1 in the main paper), i.e. the spatial origin is that suggested by Atkinson[2]. The intercept is 35.0-40.9 phonemes (as compared to 35.4-39.9 phonemes in Fig. 1). The slope is $-(4.0\text{-}8.4)\cdot 10^{-4}$ phonemes/km (as compared to $-(3.4\text{-}6.5)\cdot 10^{-4}$ phonemes/km in Fig. 1). The correlation coefficient is -0.41 (as compared to -0.32 in Fig. 1). $P < 0.001$. All intervals have been computed with 95% confidence level.

S2. Simulation models

Initially (i.e., at the onset of the out-of-Africa dispersal) there are 5 tribes (each of them with a different language) in the central cell of the grid. As explained in the main text, these are the 5 languages with less number of phonemes in our database (for simulations with a different initial condition, see Sec. S3a below). Table S1 shows the phonemes of these 5 initial languages (they have 11, 11, 13, 13 and 14 phonemes, with a total of 26 different phonemes). Every generation there is dispersal and reproduction (see the main paper for details). These features apply to all models considered in this section.

Name	# of phonemes	phonemes
PIRAHA	11	p, b, "t, k, g, ?, "s, h, i, a, "o
ROTOKAS	11	p, t, k, B, rT, g, i, "e, a, "o, u
HAWAIIAN	13	p, k, ?, m, "n, h, "l, w, i, E, a, "o, u
NASIOI	13	p, b, "t, "r[, k, m, "n, ?, i, E, a, u, o
RORO	14	p, b, tD, k, ?, m, nD, rD[, h, i, "e, a, "o, u

Table S1. Language name, number of segments and phonemes for the five initial languages. As explained in the main text, these are the languages with less number of phonemes in our database (the database is available as Supp. Info. S1). These 5 languages have a total of 26 different phonemes. In the simulation code, each language corresponds to a string of "1"s and "0"s, indicating the presence of absence of each phoneme.

We next present the results of several increasingly complex models, so that the effects due to different causes can be visualized separately. The third model below (Sec. S2c) has been used to obtain Figs. 2-3 in the main paper.

S2a. Model 1: no phonemic accumulation

In this very simple model, there is no phonemic accumulation. Thus languages do not evolve. Therefore, there are only 5 different languages in the grid, and they have 11, 11, 13, 13 and 14 phonemes (table S1).

Model 1 does not yield any geographic cline of the type detected by Atkinson[2] (see Fig. 1 in the main paper or Figs. S1-S2 above). However, this model is very useful to understand some key aspects of the simulations.

By analyzing the results of many simulation runs of this model, we have noted that, as the wave of advance of modern humans propagates, the effect of drift is so strong that a single language is selected. For example, Fig. S3 shows some snapshots from a simulation run of this model, obtained at several times after the onset of the out-of-Africa dispersal. In the first figure (t = 312 generations), we can see that the front has propagated up to about 5,000 km. As expected, near the origin of the dispersal (left-hand side), which corresponds to the center of the simulation grid, there are languages with 11, 13 and 14 phonemes. In contrast, there is a single language at distances >2,000 km along the positive horizontal axis (note that, according to table S1, there are two languages with 11 phonemes, so in principle both of them might be present at distances >2,000 km, but we have checked that this is not the case, i.e. that there is a single language at large distances). This is a spatial drift effect. It is due to the fact that not all languages in a cell disperse to *all* 4 nearest neighboring cells. For example, for the parameter values used (see the main paper), if a cell has 5 languages, one language will disperse to each of

the 4 nearest cells (i.e., along each of the 4 vertical and horizontal directions), and the fifth language will remain at the original cell. We are dealing with a grid of squared cells defined by 1,000x1,000 nodes, and we plot the results for the 500 cells from the center of the grid [i.e., the node (500,500)] to its edge along a single direction, e.g. the positive horizontal one [i.e., until the node (500,1000)]. Therefore, as the population wave of advance spreads from the center to the edges of the grid, this selection (drift) effect takes place many times. We are thus dealing with a repeated bottleneck effect. In this way, it is easy to understand intuitively that some languages do not reach the edge of the grid. This does not mean that they disappear, simply that they remain near the center of the grid (i.e. at cells with low distances from the origin), as seen in Fig. S3, and possibly spread along other directions. In the specific simulation run in Fig. S3, the language that is selected has 11 phonemes. However, this is not necessarily so, because in other simulation runs, the language selected at large distances had 13 or 14 phonemes. This is as it should, because in this very simple model there is only dispersal and reproduction, and none of both processes is related to the number of phonemes.

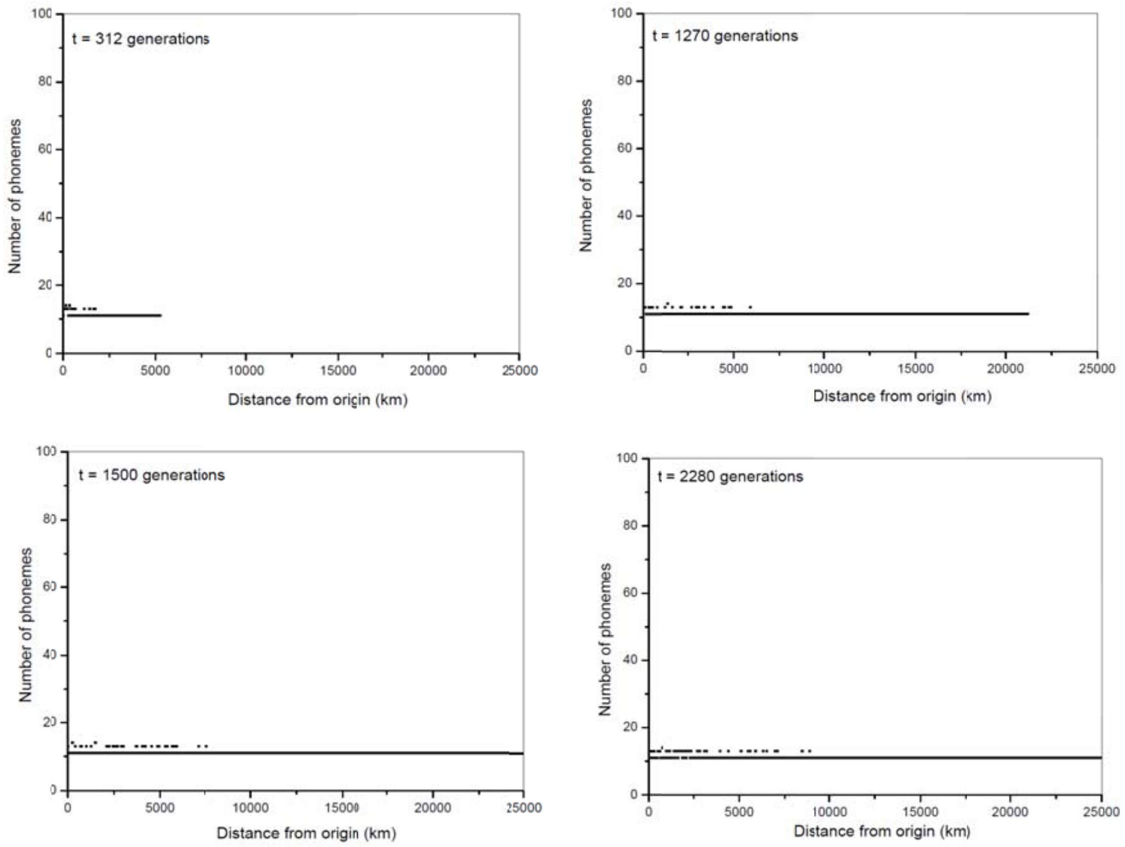

Fig. S3. An example of results from a single simulation run of model 1 (i.e., no phonemic accumulation). Note that at large distances from the origin, a single language is selected.

As expected, this first model cannot generate a cline that might correspond to that detected by Atkinson, because he detected a cline of decreasing phonemic diversity with increasing distance[2]. In contrast, in this model the language that reaches large distances does not necessarily have less phonemes (as explained above). In other words, this model generates a plot of *decreasing* phonemic diversity with increasing distance in simulation runs such that a language with few phonemes is selected (as in Fig. S3, where the selected languages has 11 phonemes). However, the same model generates a plot of *increasing* phonemic diversity with increasing distance in simulation runs such that a language with many phonemes (within the initial ones) is selected (as stated above, this happened in other simulation runs, where the language with 14 phonemes was selected). Moreover, the same model generates a plot in which phonemic diversity does not increase neither decrease much with increasing distance (this happens in simulation runs such that a language with an average number of phonemes is selected, e.g. 13 phonemes). Thus, this model cannot provide a causal explanation of the detected cline[2].

To avoid confusion, let us mention the following point. It may be argued (from Fig. S3) that this first model does generate a cline of decreasing number of phonemes *per unit area* or *per unit cell* with increasing distance. Indeed, the number of phonemes *per cell* takes into account all phonemes of all languages present in the cell considered (for example, if all 5 languages in table S1 are present in a single cell, the total number of phonemes in that cell is 26). Certainly, in Fig. S3 there are more languages, and therefore more different phonemes *per cell,* at low distances (left, 26 phonemes) than at large distances (right, 11 phonemes). However, Atkinson[2] detected a cline of decreasing number of phonemes *per language*, not *per unit area*. Therefore, for the simulations to agree with Atkinson's data, they should display a cline in the number of phonemes *per language* (not just *per unit area* or *per cell*).

S2b. Model 2: all languages accumulate phonemes

The only difference with model 1 (Sec. S2a) is that we now include a process of phonemic accumulation, as suggested by Perreault and Mathew[4]. This second model is very simple, in the sense that all languages accumulate phonemes. Again, this will not generate a cline. However, it will be useful to understand the effect of phonemic accumulation before considering a less simple model. We assume, e.g., that one phoneme is added to the language of *all* tribes in the simulation grid every 82 generations, i.e. at times t=82, 164, 246, ... generations. An example of the results of the second model is shown in Fig. S4.

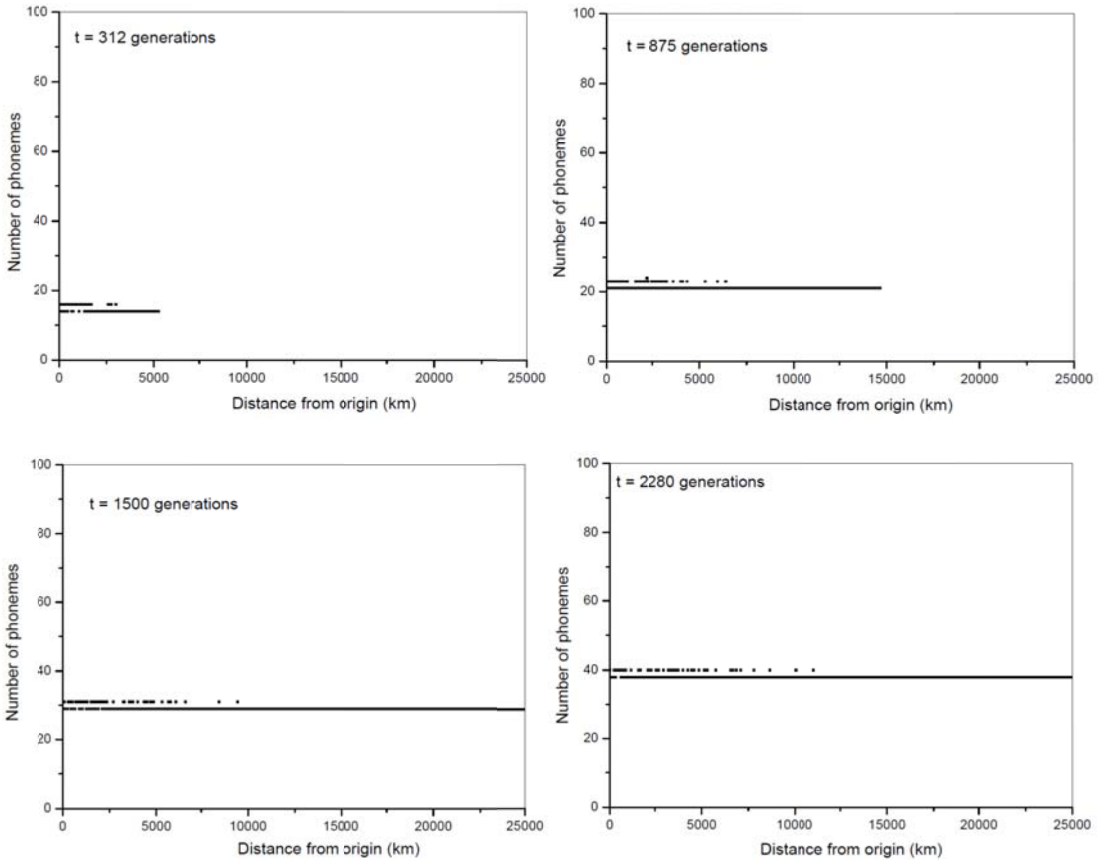

Fig. S4. An example of results from a single simulation run of model 2 (i.e., *all* languages accumulate phonemes). The accumulation rate is one phoneme every 82 generations.

It is obvious from Fig. S4 that this model does not generate a phonemic cline (as expected, because *all* languages accumulate the same number of phonemes per unit time). Fig. S4 shows how, in contrast to the previous model (Fig. S3), the number of phonemes gradually increases with time. We can understand this dynamics quantitatively as follows. The accumulation rate is one phoneme every 82 generations. Thus, after 2,280 generations, we expect the addition of 2,280/82=27 phonemes. Adding up the initial number (11-14 phonemes), we obtain 38-41 phonemes, which is consistent with the range obtained in Fig. S4 for t=2,280 gen.

This model, as the previous one, will make it easier to understand the more complicated models that are discussed in the two next subsections.

S2c. Model 3: only languages with high speaker *densities* accumulate phonemes

Model 3 has been used to obtain the simulation results reported in the main paper (Figs. 2, 3). In this model, languages mutate (i.e., accumulate phonemes) only in regions (cells) where the population density of modern humans is large enough. For definiteness, both in the main paper and here we consider mutations only if the population has reached carrying capacity, i.e. only in cells with N=5 different tribes (see Sec. S3g for simulations if languages mutate in cells with N≥4 different tribes, and Sec. S3b for simulations with a different value of N).

Note that in this model, if a cell has carrying capacity (i.e., if it has 5 tribes), *all languages* in it accumulate phonemes, irrespectively on whether all those 5 languages are the same or not. In other words, in a cell at carrying capacity (5 tribes), we can have 1, 2, 3, 4 or 5 different languages, and in this model all of them accumulate phonemes (but the results are similar if phonemic accumulation is switched on at a population density threshold that is lower than the saturation density, see Sec. S3g). Admittedly, the possibility that phonemic accumulation increases with population density is an assumption and we have no proof for it, but previous work on cultural and linguistic evolution has repeatedly proposed that culture accumulation increases with population density (for some theoretical and empirical references, see the main paper, end of Sec. 2). In any case, our aim is to see whether under this assumption a cline is generated or not. Of course, if a cline is generated, this will not proof that this model is correct, but only that it is one possible mechanism that deserves further investigation (and comparison to other viable mechanisms that may be found in future work). Overall, we think that our work is only a first step showing the usefulness of simulation approaches to analyze quantitatively the proposal of a serial founder effect of language expansion from Africa[2], in the sense of showing if is possible to generate a cline from clear assumptions, as well as to compare it to the observed cline.

For later discussion, it is important to note that in saturated cells (i.e., cells where the population density is maximum), the 5 languages have the same number of speakers *per unit area,* irrespectively on whether all 5 tribes speak the same language or not. For example, if all 5 tribes speak the same language, the area occupied by it is the cell size (namely 50km x 50km). If 4 tribes speak a language and 1 tribe a different one, the first has 4/5 of the cell speakers but also 4/5 of the cell area, and the second one has 1/5 of the cell speakers but also 1/5 of the cell area. Thus the 5 languages have the same number of speakers *per unit area,* irrespectively on whether all 5 tribes speak the same language or not. Therefore, in this model a language accumulates phonemes only if its *density* of speakers is high enough. Moreover, a high population density (e.g., 5 tribes per cell) corresponds to a high speaker density (1 tribe or 400 speakers per 500 km^2), and a low population density (e.g., 1 tribe per cell) corresponds to a low speaker density (400 speakers/2,500 km^2).

Before discussing the results of this third model, let us mention that we have also tested another model in which the accumulation of phonemes does not depend on the speaker *density* but on the *number* of speakers. However, that model did not generate a cline consistent with the observed one (see Sec. S2d below for details).

Figure S5 shows an example of a simulation run of model 3 (i.e., the same model as that used to obtain the results in the main paper, namely Figs. 2 and 3). It is seen that, in contrast to the two previous models (Secs. S3a-b), a phonemic cline is generated. We can understand the reason as follows. As in model 2 (Sec. S3b), one phoneme is added every 82 generations, i.e. at t=82, 164, 246, ... generations. However, unlike model 2, this does not happen for all languages, but only for those in cells (or regions) where the population density is equal to its maximum possible value (N=5). Therefore, in regions where some populations have arrived but the population density has not reached saturation yet (i.e., in cells with N≤5), phonemes are not accumulated. Such cells correspond to the pioneering populations of modern humans, i.e. to the front of the wave of advance. In Fig. S5, this corresponds to about 5,000 km in the first plot (t=312 gen) and about 22,000 km in the second plot (t=1270 gen). Thus languages on the front tend to accumulate few phonemes, and populations speaking these low-diversity languages disperse, reproduce, and propagate the front further away. This is in sharp contrast with regions near the origin (left-hand side of the plots), that lie behind the front and are therefore less affected by this process. This is the reason why model 3 generates a cline of decreasing number of phonemes per language with increasing distance from the origin of the dispersal of modern humans (Fig. S5 and Figs. 2, 3 in the main paper). Because this point is rather important, we also explain it graphically in Fig. S6.

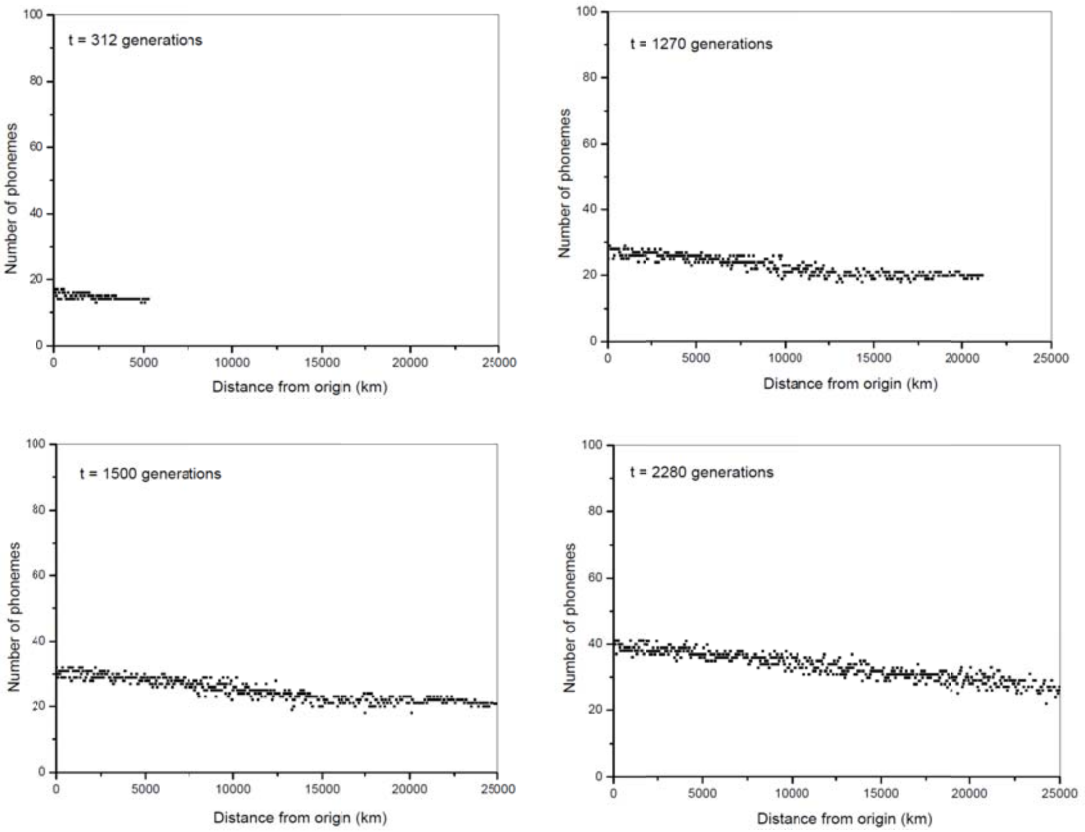

Fig. S5. An example of results from a single simulation run of model 3 (i.e., only languages with a high enough *density* of speakers accumulate phonemes). The accumulation rate is one phoneme every 82 generations. The last plot (t=2280 gen) is the same as Fig. 3 in the main paper.

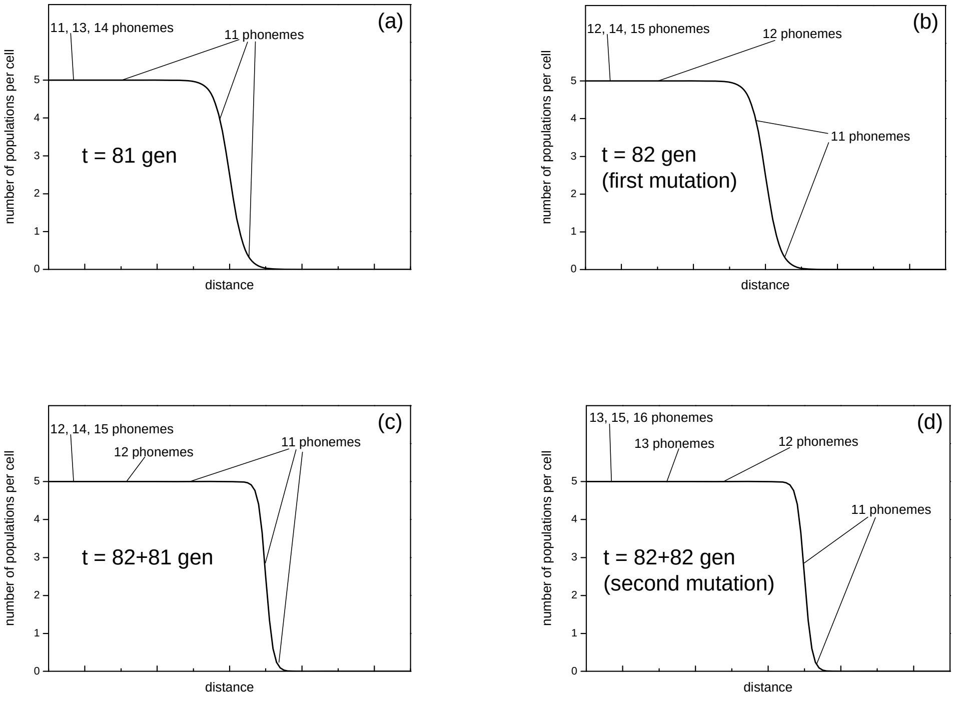

Fig. S6. This figure is a representation of some results from model 3. It is very useful to explain in detail how the cline (Fig. S5) is generated. Note that here the vertical axis is the number of *tribes* per cell (not the number of *phonemes* per language as in Fig. S5). Initially we have languages with 11, 13 and 14 phonemes in the center of the grid (i.e., at small distances). Those tribes disperse and reproduce, and drift selects a single language in the cells located at large enough distances from the center (see Sec. S2a for details). This is shown again in (a), where (for clarity) the selected language has 11 phonemes. At time t=82 gen (b), cells at saturation (i.e., with 5 tribes, left-hand side of (a)) add a phoneme, whereas cells with less than 5 tribes (right side of (a)) do not. At time 82+81 gen=163 gen (c), front propagation and drift have led to language selection again. At time t=82·2 gen=164 gen (d), languages behind the front accumulate a second phoneme, whereas languages on the front do not. This repeated bottleneck effect repeats many times, and gives rise to a cline of decreasing number of phonemes per language as a function of distance from the origin of the dispersal (Fig. S5). For the sake of clarity, to some extent these plots oversimplify the process, in the sense that according to them, tribes on the front would always have 11 phonemes, whereas in practice some phonemes are accumulated on the front, as seen in Fig. S5 for t=312 gen and t=1270 gen (the reason is that the saturated cells with highest distances are very close to cells that are not empty but still below saturation, and dispersal takes place between them). But the important point is that phonemic accumulation is weaker in the front than near the origin, for the reasons illustrated by the plots in this figure.

In Fig. S5, all languages in cells at saturation density (i.e., cells with N=5 tribes of 400 individuals each, see the main paper) accumulate phonemes, and no languages in the rest of cells

accumulate any phonemes. In fact, it is not necessary that the accumulation threshold is equal to the population saturation density (N=5). For example, if we assumed that all languages in cells with N≥4 populations accumulate phonemes, a similar cline would also appear (see Sec. 3g).

S2d. Model 4: only languages with large *numbers* of speakers accumulate phonemes

For the sake of completeness and clarity, we would like to summarize an additional model. As models 1 and 2, model 4 has *not* been used in the main paper and it cannot explain Atkinson's observed cline. However, it further clarifies the conditions under which such a cline can arise.

All of the three models above, as well as this fourth one, share some features explained in the main paper: (i) each cell can have up to N=5 tribes (see Sec. S3b for simulations with a different value of N); (ii) a single language in spoken by each of these tribes; (iii) the languages spoken by the tribes in each cell are not necessarily different.

In model 3 (previous subsection and main paper), if a cell has carrying capacity (i.e., if it has 5 tribes), *all languages* in it accumulate phonemes, irrespectively on whether all those 5 languages are the same, all of them are different, etc. In other words, we can have 1, 2, 3, 4 or 5 different languages, and all of them accumulate phonemes (if the cell has N=5 tribes). In contrast, in model 4 for each cell, only languages spoken by at least N* tribes accumulate phonemes. Obviously the possible values of N* are N*=1,2,3,4,5 (and the case N*=1 corresponds to the second model, Sec. S2b). Thus, if N*>1 only languages with a large enough population of speakers in each region (cell) accumulate phonemes. Below we show results for N*=3, but they are similar for other values of N*.

In this model, a language mutates (i.e., accumulates phonemes) only if it has a high enough population *number* of speakers (whereas in model 3, it did so only if had a high enough population *density* of speakers). We can see this as follows. Consider first an example such that a saturated cell (5 tribes) has 3 tribes with one language and 2 tribes with another language. In model 4 the language of the first 3 tribes will mutate, but the languages of the other 2 tribes will not (in model 3, in contrast, all 5 languages mutate). Since each tribe has 400 speakers and each cell has 2,500 km^2 (see the main paper), all of these 5 tribes have the same speaker density (namely, 400 speakers/500 km^2=0.8 speakers/km^2) but they have different numbers of speakers (1,200 speakers for the first language and 800 speakers for the second). As a second example, consider a cell with 3 tribes. They will not mutate in model 3. However, if all of them have the same language, they will mutate in model 4 (because this language is spoken by 3 tribes or 1,200 speakers). The speaker density (1,200 speakers/2,500 km^2=0.48 speakers/km^2) of this language is lower than in the first example. These examples show that phonemic

accumulation is related to the *density* of speakers in model 3 and to the *number* of speakers (in the region or cell considered) in model 4.

We show results from model 4 in Fig. S7. In this figure we see that in model 4 there is a cline, but it is opposite to the observed one: the simulations yield an increase for the number of phonemes with increasing distance from the origin of the dispersal (Fig. S7), whereas the observed data display a decrease (Figs. 1, S1 and S2). This result is related to the fact that, while the wave of advance propagates, spatial drift leads to the selection of a single language at large distances. This has been explained in detail in the first model (Sec. S2a). In that model, this effect was clearer due to the absence of phonemic accumulation (Fig. S3). However, we can again see the same effect in Fig. S7, before the front reaches the end of the grid: in the first plot (t=312 gen), we see that the occupied cells to the right (at about 4,000-5,500 km) have a single language. The same is seen in the second plot in Fig. S7 (t=1,270 gen), albeit less clearly because in this case less cells have the same language (only those at about 21,000-21,500km), due to the fact that less time has passed since the last phoneme was added: the second plot in Fig. S7 has been obtained at t=1,270 gen/82=15.49 mutations, so that only 0.49·82=40 generations have passed since the last mutation (or phonemic accumulation event), compared to the first plot in Fig. S7, which has been obtained at t=312 gen/82=3.8 mutations, so that 0.8·82=66 generations have passed since the last mutation. The key point is simply that during each time interval (namely, 82 generations) from the accumulation of the last phoneme until the accumulation of the next one, a single language will be selected at large distances. Hence, cells *at large distances* will all have the same language. If such a cell has 3 or more tribes, a new phoneme will be added to their language. In contrast, in the cells close to the origin of the dispersal (*low distances*, left of the plots in Fig. S7) we see from the plots that there are several languages. For example, consider a saturated cell (5 tribes) with 3 languages, present in 2, 2 and 1 tribes. None of those will add a new phoneme because in each cell, languages spoken by less than N*=3 tribes do not add a phoneme (in model 4). This is why in the plots in Fig. S7, tribes at *low distances* remain with few phonemes, whereas tribes at *large distances* accumulate phonemes. This explains why a cline of increasing phonemic diversity is obtained (Fig. S7), contrary to the observations (Figs. 1, S1 and S2).

This model clarifies the joint effect of drift and phonemic accumulation. It also shows that the observed cline cannot be explained by a simple model based on assuming that the phonemic accumulation rate is faster for languages with a large *number* of speakers in the cell or region considered.

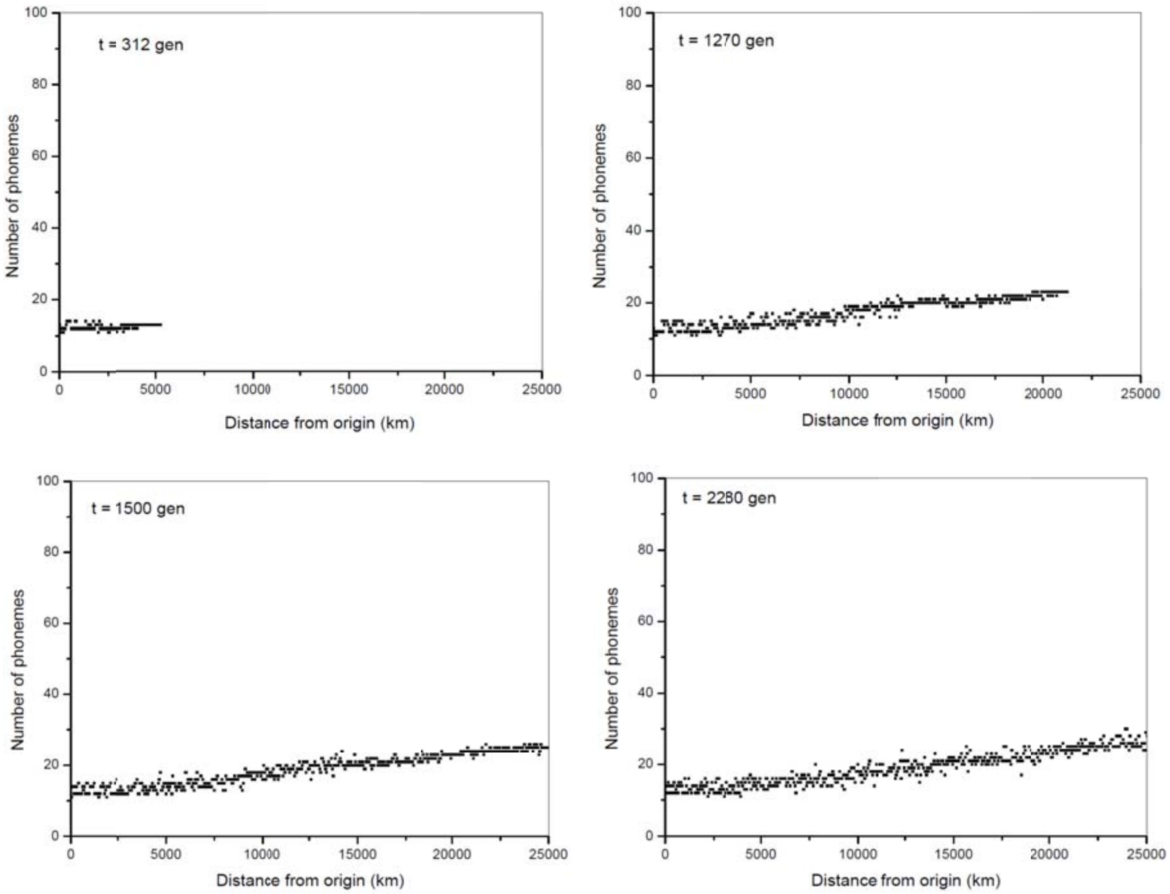

Fig. S7. An example of results from a single simulation run of model 4 (i.e., only languages with large *numbers* of speakers accumulate phonemes). The accumulation rate is one phoneme every 82 generations.

S3. Sensitivity analysis

This section contains a sensitivity analysis of the simulation results (Figs. 2-3) in the main paper.

S3a. Number of initial languages

The simulations in the main paper assume that, at the onset of the out-of-Africa dispersal, there were 5 tribes at the central node of the simulation grid, and each of these 5 tribes spoke a different language, with phonemic diversity equal to that of one of the present 5 languages with lowest diversities (table S1). We repeated the simulations by assuming, instead, that all 5 tribes spoke the same language at the onset of the out-of-Africa dispersal, with diversity equal to that of the present languages with lowest phonemic diversity, i.e. 11 phonemes (table S1, Piraha or Rotokas). The results are shown in the last two rows in table S2. Each intercept (which gives the average number of present languages at the origin of the dispersal) is lower than the

corresponding intercept with 5 initial languages. This is as expected, because the initial number of phonemes with a single initial language (11) is lower that with 5 initial languages (11-14 phonemes). The intercepts with a single initial language imply a range 29-38 phonemes for languages spoken today in Africa, which is consistent with the observed one (35-40 phonemes, from Fig. 1 or the first row in table S2). On the other hand, we have no intuitive explanation for the effect of the number of initial languages on the slope. However, the range for the slope obtained from the single-language models (-(1.2-5.9)·10^{-4} phonemes/km) is consistent with the observed slope (-(3.4-6.5)·10^{-4} phonemes/km). Thus the conclusions are the same as for the models in the main paper (table S2, rows 2-3).

	Slope (phonemes/km)	Intercept (phonemes)	*r*	*n*
Observed (Fig. 1)	-(3.4-6.5)·10^{-4}	35.4-39.9	-0.317	366
5 languages, 0.26/ky (Fig. 2)	-(1.4-1.7)·10^{-4}	31.2-31.6	-0.671	501
5 languages, 0.38/ky (Fig. 3)	-(5.1-5.5)·10^{-4}	39.5-40.1	-0.929	501
1 language, 0.26/ky	-(1.2-1.5)·10^{-4}	29.1-29.5	-0.626	501
1 language, 0.38/ky	-(5.5-5.9)·10^{-4}	37.7-38.2	-0.941	501

Table S2. Slope and intercepts for several models, reported with 95% confidence-level intervals. *n* is the number of languages used in the regression and in Figs. 2-3 in the main paper (we have used one language per cell, chosen at random, which substantially increases the clarity of the figures).

S3b. Number of tribes per node

The simulations in the main paper use a square grid of nodes with up to 5 tribes (N=5) per squared cell (each cell has an area of 50km·50km=2,500 km^2). In the main paper, this maximum value N=5 tribes/node has been estimated as follows. Hassan[5] gathered population densities of hunter-gatherers and they vary widely, so we use an intermediate value of 0.8 people/km^2 (this is representative of values reported for populations in various continents, such as the Ituri pygmies (Africa), the Andaman Islanders (Asia), and Californian hunter-gatherers). Since the cell centered at each node of our grid has 50·50=2,500 km^2, this population density leads to about 2,000 people/node. A tribe is usually defined as a reproductive (i.e., highly endogamous) group with a common language, possibly spoken also by other tribes[6]. A representative value for the population size of a tribe is about 400 people[7]. These values imply that each node can have up to N=5 tribes. In order to see to what extent the results might depend on these ethnographic estimates, we note that some estimations of population densities of hunter-gatherers[5] are about 2-3 people/km^2. Similarly, tribe numbers of about 500-600 people/tribe have been reported[8]. If we repeat the calculations above with a population density of 2.4 people/km^2 and a tribe size of 600 individuals, we obtain N=10 tribes. Thus we here report the results of the

same simulations as in the main paper, but assuming that each node can have up to N=10 tribes (instead of N=5). Therefore, we now consider 10 tribes at the onset of the out-of-Africa dispersal, with 2 tribes for each of the 5 languages used in the main paper (table S1), in order to use the same initial phonemic diversity. The results are shown in the last two rows in table S3. The intercepts imply a range 31-39 phonemes for languages spoken today in Africa, which is consistent with the observed range (35-40 phonemes, from Fig. 1 or the first row in table S3). We also note that the slopes of the N=10 models imply a range ($-(2.6\text{-}3.4)\cdot10^{-4}$ phonemes/km) that is marginally consistent with the observed slope (first row in table S3). Thus the conclusions of the main paper remain essentially the same.

	Slope (phonemes/km)	Intercept (phonemes)	*r*	*n*
Observed (Fig. 1)	$-(3.4\text{-}6.5)\cdot10^{-4}$	35.4-39.9	-0.317	366
N = 5, 0.26/ky (Fig. 2)	$-(1.4\text{-}1.7)\cdot10^{-4}$	31.2-31.6	-0.671	501
N = 5, 0.38/ky (Fig. 3)	$-(5.1\text{-}5.5)\cdot10^{-4}$	39.5-40.1	-0.929	501
N = 10, 0.26/ky	$-(2.6\text{-}2.7)\cdot10^{-4}$	31.1-31.5	-0.855	501
N = 10, 0.38/ky	$-(3.1\text{-}3.4)\cdot10^{-4}$	38.6-39.1	-0.889	501

Table S3. Slope and intercepts for several models, reported with 95% confidence-level intervals. *n* is the number of languages used in the regression and in Figs. 2-3 in the main paper (we have used one language per cell, chosen at random, which substantially increases the clarity of the figures).

Let us note that neither the *population density* nor the *tribe population size* are used in the simulations, but only their quotient multiplied by the area of each square in the simulation grid. This yields N (the number of tribes per node), which is used in the simulations. Therefore, the simulation results (Figs. 2-3 in the main paper and tables in this Supp. Info.) are valid for many other values of the population density and tribe population size, as far as their quotient is the same. For instance, Figs. 2-3 in the main paper have been computed for a population density of 0.8 people/km^2 and a tribe population size of 400 people (leading to N=5), but they are exactly the same for, e.g., 0.08 people/km^2 and 40 people/tribe (leading again to N=5), etc. In the case of groups of only 40 people, however, we would be dealing with bands rather than tribes[9].

The distance between two neighboring lattice nodes ($d = 50$ km in the main paper but varied in Sec. S3f) determines the area of each square in the simulation grid, d^2. In fact, neither the value of the distance d nor that of the area d^2 are used in the simulations. However, the value of d is necessary to plot phonemic diversity against distance (measured in km), as in Figs. 2-3 in the main paper, and therefore to compute the linear fits (tables in this Supp. Info).

S3c. Time elapsed from the onset of the out-of-Africa dispersal

The simulations in the main paper assume that the onset of the out-of-Africa dispersal took place 70 ky ago. We repeated the simulations assuming that the dispersal out of Africa begun 40 ky ago, and also assuming it begun 100 ky ago (in both cases, we used the same initial set of 5 languages and all other parameter values as in the simulations leading to Figs. 2-3). The results are reported in table S4.

Had the dispersal begun recently (40 ky ago), there would have been less time for phonemes to accumulate. This is why the intercepts imply a range for the number of phonemes of present African languages (21-27 phonemes, rows 4-5 in table S4) that is lower than in the main paper simulations, Figs. 2-3 (31-40 phonemes, rows 2-3 in table S4). The range from simulations beginning 40 ky ago (21-27 phonemes) is also lower than the observed one (35-40 phonemes, row 1 in table S4). Thus the observed phonemic diversity cline is inconsistent with such a recent out-of-Africa dispersal. This is not a problem because 40 ky ago is too late according to the archaeological and genetic data available at present, which indicate a likely date of 58-87 ky ago[10,11]. In fact, such a recent dispersal would be surprising also from a purely ethnographic perspective, because in our models the front reaches the end of the grid (i.e., distances similar to the largest distances from Atkinson's best-fit origin in the linguistic dataset) at about 1,470 gen or 47 ky (Fig. S5), i.e. later than 40 ky after the start of the out-of-Africa dispersal (this is also why the number of languages *n* in table S4 is lower for t=40 ky).

It is true, however, that there is debate on possibly older dates for the exit out of Africa[12]. Thus we repeated the simulation also for a very early dispersal (100 ky). Then more phonemes accumulate, as expected (the intercept is 37-51 phonemes, from the last two rows in table S4). This range is consistent the observed one (35-40 phonemes).

We note form table S4 that the slopes from both models, namely $-(1\text{-}4)\cdot 10^{-4}$ phonemes/km (t = 40 ky) and $-(3\text{-}5)\cdot 10^{-4}$ phonemes/km (t = 100 ky), are consistent with the observed slope, i.e. $-(3\text{-}7)\cdot 10^{-4}$ phonemes/km.

	Slope (phonemes/km)	Intercept (phonemes)	*r*	*n*
Observed (Fig. 1)	$-(3.4\text{-}6.5)\cdot 10^{-4}$	35.4-39.9	-0.317	366
t = 70 ky, 0.26/ky (Fig. 2)	$-(1.4\text{-}1.7)\cdot 10^{-4}$	31.2-31.6	-0.671	501
t = 70 ky, 0.38/ky (Fig. 3)	$-(5.1\text{-}5.5)\cdot 10^{-4}$	39.5-40.1	-0.929	501
t = 40 ky, 0.26/ky	$-(1.4\text{-}1.8)\cdot 10^{-4}$	21.2-21.7	-0.616	419
t = 40 ky, 0.38/ky	$-(3.9\text{-}4.4)\cdot 10^{-4}$	26.1-26.7	-0.862	419

t = 100 ky, 0.26/ky	$-(2.9\text{-}3.2)\cdot 10^{-4}$	37.4-37.8	-0.856	501
t = 100 ky, 0.38/ky	$-(4.8\text{-}5.2)\cdot 10^{-4}$	50.1-50.7	-0.926	501

Table S4. Slope and intercepts for several models, reported with 95% confidence-level intervals. *n* is the number of languages used in the regression and in Figs. 2-3 in the main paper (we have used one language per cell, chosen at random, which substantially increases the clarity of the figures).

S3d. Generation time

In the main paper we have recalled that in reaction-dispersal modelling, the mean parent-children age difference should be used (not the difference for the eldest child)[13]. The simulations in the main paper assume that the generation time (defined as the mean age difference between a parent and his/her children) is T =32 y (this value has been used for preindustrial populations previously[13,14]). In order to estimate a range for *T,* rather than a single value, and taking care that we are dealing with hunter-gatherers, we note that the generation time of hunter-gatherers is about 2 y longer than for farmers[15] and that the generation time for the latter (as defined above) has been estimated from ethnographic data[13] as 27-36 y. Thus we repeated the simulations assuming T =29 y and T =38 y but keeping the onset of the out-of-Africa dispersal at 70 ky ago (so that the simulations run for 2,144 and 1,843 generations, respectively, rather than 2,280 generations as in the main paper). All other parameter values were the same as in the main paper, including the lower and upper bounds for the phonemic accumulation rate estimated by Perreault and Mathew[4], namely 0.26 and 0.38 phonemes/ky. Thus for T =29 y we respectively added a phoneme every 133 and 91 generations, and for T =38 y we respectively added a phoneme every 101 and 69 generations (instead of, respectively, every 120 and 82 generations, as in the main paper (T =32 y)).

In table S5 we compare the observed phonemic cline (Fig. 1) with that obtained from the models in the main paper (Figs. 2-3) and from the two additional models (T =38 y and T =29 y). The intercepts (present African phonemic diversity) from the simulations with T =38 y (30-37 phonemes) and with T =29 y (29-37 phonemes) are both consistent with the observed range (35-40 phonemes). On the other hand, the slope range turns out to be the same for T = 38 y and T = 29 y, namely -(3-4)·10-4 phonemes/km (table S5), which is consistent with the observed range, i.e. $-(3\text{-}7)\cdot 10^{-4}$ phonemes/km (first row in table S5). Thus for a wide range of values of the generation time, the intercept and slope of the present cline remain within the observed range.

	Slope (phonemes/km)	Intercept (phonemes)	*r*	*n*
Observed (Fig. 1)	$-(3.4\text{-}6.5)\cdot 10^{-4}$	35.4-39.9	-0.317	366

T =32 y, 0.26/ky (Fig. 2)<br>T =32 y, 0.38/ky (Fig. 3)	$-(1.4\text{-}1.7)\cdot 10^{-4}$<br>$-(5.1\text{-}5.5)\cdot 10^{-4}$	31.2-31.6<br>39.5-40.1	-0.671<br>-0.929	501<br>501
$T = 38$ y, 0.26/ky<br>$T = 38$ y, 0.38/ky	$-(4.1\text{-}4.4)\cdot 10^{-4}$<br>$-(3.3\text{-}3.6)\cdot 10^{-4}$	30.3-30.8<br>36.6-37.2	-0.911<br>-0.868	501<br>501
$T = 29$ y, 0.26/ky<br>$T = 29$ y, 0.38/ky	$-(3.7\text{-}4.1)\cdot 10^{-4}$<br>$-(3.0\text{-}3.4)\cdot 10^{-4}$	29.3-29.8<br>36.0-36.5	-0.904<br>-0.851	501<br>501

Table S5. Slope and intercepts for several models, reported with 95% confidence-level intervals. *n* is the number of languages used in the regression and in Figs. 2-3 in the main paper (we have used one language per cell, chosen at random, which substantially increases the clarity of the figures).

S3e. Initial growth rate

The simulations reported in the main paper use a growth rate ($a = 0.01$ y^{-1}) that was previously used by Conolly and co-workers[16]. They estimated it as a representative average using several archaeological datasets. However, the initial growth rate of human populations can be substantially higher in low-density populations. For example, the value 0.017 y^{-1} for hunter-gatherers has been estimated[14]. We have thus repeated the simulations with $a = 0.017$ y^{-1} and all other parameter values as in the main paper. This leads to $R_0 = e^{aT} \approx 1.7$, so we implement net reproduction simply by generating new additional tribes such that the final number of tribes at the node considered is the nearest integer to 1.7 times the initial number, respectively (instead of 1.4 times the initial number, as in the main paper).

We note from table S6 that the intercept range is 31-40 phonemes ($a = 0.017$ y^{-1}), which is consistent with the observed range (35-40 phonemes). The slope range is $-(3\text{-}7)\cdot 10^{-4}$ phonemes/km ($a = 0.017$ y^{-1}), which agrees with the observed range, i.e. $-(3\text{-}7)\cdot 10^{-4}$ phonemes/km (first row in table S6). Thus for this higher initial growth rate of the population, the simulated cline is still consistent with the observed one.

	Slope (phonemes/km)	Intercept (phonemes)	*r*	*n*
Observed (Fig. 1)	$-(3.4\text{-}6.5)\cdot 10^{-4}$	35.4-39.9	-0.317	366
$a = 0.01$ y^{-1}, 0.26/ky (Fig. 2)<br>$a = 0.01$ y^{-1}, 0.48/ky (Fig. 3)	$-(1.4\text{-}1.7)\cdot 10^{-4}$<br>$-(5.1\text{-}5.5)\cdot 10^{-4}$	31.2-31.6<br>39.5-40.1	-0.671<br>-0.929	501<br>501
$a = 0.017$ y^{-1}, 0.26/ky<br>$a = 0.017$ y^{-1}, 0.38/ky	$-(3.2\text{-}3.6)\cdot 10^{-4}$<br>$-(6.7\text{-}7.1)\cdot 10^{-4}$	31.4-31.8<br>39.0-39.6	-0.884<br>-0.945	501<br>501
$a = 0.027$ y^{-1}, 0.26/ky<br>$a = 0.027$ y^{-1}, 0.38/ky	$(-8.1\text{-}7.1)\cdot 10^{-6}$<br>$-(7.7\text{-}9.6)\cdot 10^{-5}$	32.8-32.6<br>38.1-38.4	-0.006<br>-0.625	501<br>501

Table S6. Slope and intercepts for several models, reported with 95% confidence-level intervals. *n* is the number of languages used in the regression and in Figs. 2-3 in the main paper (we have used one language per cell, chosen at random, which substantially increases the clarity of the figures).

In some cases, growth rates for hunter-gatherers as high as $a = 0.027$ y^{-1} ($R_0 = 2.4$) have been considered[14]. If the population reproduces so fast (last two rows in table S6), it turns out that the slope of the cline is too small to be consistent with the observed one (first row).

S3f. Mobility behavior

In the main paper we have used a square grid on nodes such that the distance between any two first neighbors is *d* = 50 km, because this is the characteristic dispersal distance per generation of some pre-industrial populations. It was estimated[17] from the formula $m = d^2 \cdot (1 - p_e)$, where m=1544 km^2/gen is the mobility (mean-squared displacement per generation) and p_e=0.38 is the persistence (fraction of the population that does not move). However, some hunter-gatherer populations reported in the ethnographic literature have substantially larger values of *d*, e.g. *d* = 68 km (Bofi-Aka[18]) and even *d* = 75 km (Aka[18]). Therefore, in this subsection we report additional results from the simulations, obtained for values of *d* and p_e characteristic of the Aka[18], namely *m* = 3852 km^2/gen and p_e = 0.31, which imply that *d* = 75 km. The dimensions of the simulation lattice were the same as in the main paper (i.e., 25,000 km from the center to the edges) and we computed the number of nodes along the positive horizontal direction accordingly (i.e., by dividing 25,000 km by *d*). This yields less nodes for *d* = 75 km than for *d* = 50 km, and is the reason of the lower value of *n* in table S7 for the Aka (as compared to *n*=501 for *d* = 50 km).

We note from table S7 that the intercept range for the Aka (30-40 phonemes) is consistent with the observed range (35-40 phonemes). The slope range for the Aka is -(3-4)·10^{-4} phonemes/km, also consistent with the observed range (namely -(3-7) · 10^{-4} phonemes/km). Therefore, also for values of *d* and p_e obtained from a real example (Aka) with high mobility, the simulated cline is consistent with the observed one.

	Slope (phonemes/km)	Intercept (phonemes)	*r*	*n*
Observed (Fig. 1)	-(3.4-6.5)·10^{-4}	35.4-39.9	-0.317	366
$d = 50$ km, p_e = 0.38, 0.26/ky (Fig 2)	-(1.4-1.7)·10^{-4}	31.2-31.6	-0.671	501
$d = 50$ km, p_e = 0.38, 0.38/ky (Fig 3)	-(5.1-5.5)·10^{-4}	39.5-40.1	-0.929	501

Aka (d = 75 km, p_e = 0.31), 0.26/ky<br>Aka (d = 75 km, p_e = 0.31), 0.38/ky	$-(3.0\text{-}3.4)\cdot10^{-4}$<br>$-(3.8\text{-}4.4)\cdot10^{-4}$	30.1-30.4<br>39.7-40.2	-0.895<br>-0.858	334<br>334

Table S7. Slope and intercepts for several models, reported with 95% confidence-level intervals. *n* is the number of languages used in the regression and in Figs. 2-3 in the main paper (we have used one language per cell, chosen at random, which substantially increases the clarity of the figures).

S3g. Demographic threshold of phonemic accumulation

In the main paper (and Sec. S2c) we have assumed that, in cells at saturation density (i.e., cells with N=5 tribes of 400 individuals each), all languages accumulate phonemes, and that no languages in the rest of cells accumulate any phonemes. However, it is not necessary to assume that the accumulation threshold (i.e., the speaker density above which languages accumulate phonemes) is equal to the population saturation density (N=5). In the last two rows of table 8 we show the results for a threshold of N=4, i.e. if all languages in cells with N≥4 tribes accumulate phonemes. It is seen that both the slope ($-(2\text{-}6)\cdot10^{-4}$ phonemes/km) and the intercept (32-39 phonemes) of the simulated cline are again consistent with those of the observed one (first row in table S8). Thus the conclusions of the main paper are the same.

	Slope (phonemes/km)	Intercept (phonemes)	*r*	*n*
Observed (Fig. 1)	$-(3.4\text{-}6.5)\cdot10^{-4}$	35.4-39.9	-0.317	366
threshold N=5, 0.26/ky (Fig. 2)<br>threshold N=5, 0.38/ky (Fig. 3)	$-(1.4\text{-}1.7)\cdot10^{-4}$<br>$-(5.1\text{-}5.5)\cdot10^{-4}$	31.2-31.6<br>39.5-40.1	-0.671<br>-0.929	501<br>501
threshold N=4, 0.26/ky<br>threshold N=4, 0.38/ky	$-(2.3\text{-}2.6)\cdot10^{-4}$<br>$-(5.2\text{-}5.6)\cdot10^{-4}$	31.6-32.2<br>38.4-38.9	-0.800<br>-0.933	501<br>501

Table S8. Slope and intercepts for several models, reported with 95% confidence-level intervals. *n* is the number of languages used in the regression and in Figs. 2-3 in the main paper (we have used one language per cell, chosen at random, which substantially increases the clarity of the figures).

Finally, it is important to stress that the computer program uses random numbers (see main paper, Methods). For this reason, when a simulation is repeated, the results (tables S2-S8) are not exactly the same.

References

1. Creanza, N., Ruhlen, M., Pemberton, T.J., Rosenberg, N.A. & Feldman, M.W., Ramachandran S. A comparison of worldwide phonemic and genetic variation in human populations. *Proc. Natl. Acad. Sci. U.S.A.* **112**, 1265-1272 (2015).

2. Atkinson, Q. D. Phonemic diversity supports a serial founder effect model of language expansion from Africa. *Science* **332**, 346–349 (2011a).

3. Hunley, K., Bowern, C. & Healy, M. Rejection of a serial founder effects model of genetic and linguistic evolution. *Proc. R. Soc. B* **279**, 2281-2288 (2012).

4. Perreault, C. & Mathew, S. Dating the Origin of Language Using Phonemic Diversity. *PLoS One* **7**, e35289 (2012).

5. Hassan, F. A., *Demographic archaeology* (Academic Press, New York, 1981), table 2.1.

6. Dixon, R. M. W. Tribes, languages and other boundaries in northeast Queensland. In *Tribes and boundaries in Australia,* ed. by P. Nicolas (Australian institute of aboriginal studies, Canberra, 1976), pp. 207-238.

7. Lee, R. B. & deVore, I., eds. *Man the hunter* (Aldine, Chicago, 1968).

8. Flood, J. Man and ecology in the highlands of southeastern Austrialia: a case study. In Tribes, languages and other boundaries in northeast Queensland. In *Tribes and boundaries in Australia,* ed. by P. Nicolas (Australian institute of aboriginal studies, Canberra, 1976), pp. 30-49.

9. Henn, B. M., Cavalli-Sforza, L.L. & Feldman, M.W. The great human expansion. *Proc. Natl. Acad. Sci. U.S.A.* **109**, 17758-17764 (2012).

10. Oppenheimer, S. Out-of-Africa, the peopling of continents and islands: tracing uniparental gene trees across the map. *Phil. Trans. R. Soc. B* **367**, 770-784 (2012).

11. Mellars, P., Gori, K. C., Carr, M., Soares, P. A. & Ricards, M. B. Genetic and archaeological perspectives on the initial modern human colonization of southern Asia. *Proc. Natl. Acad. Sci. U.S.A.* **110**, 10699-10704 (2013).

12. Petraglia, M. D., Haslam, M., Fuller, D. Q., Boivin, N. & Clarkson, C. Out of Africa: new hypotheses and evidence for the dispersal of Homo sapiens along the Indian Ocean rim. *Ann. Hum. Biol*. 37, 288-311 (2010).

13. Fort, J., Jana, D., Humet, J. M. Multidelayed random walks: theory and application to the Neolithic transition in Europe. *Phys. Rev. E*. **70**, 031913 (2004), especially note [24].

14. Fort, J., Pujol, T. & Cavalli-Sforza, L. L. Palaeolithic populations and waves of advance. *Cambridge Archaeol. J.* **14**, 53-61 (2004).

15. Ammerman, A.J. & Cavalli-Sforza, L. L. The Neolithic transition and the Genetics of populations in Europe (Princeton University Press, Princeton, 1984).

16. Conolly, J., Colledge, S. & Shennan, S. Founder effect, drift, and adaptive change in domestic crop use in early Neolithic Europe. *J. Arch. Sci.* **35**, 2797-2804 (2008).

17. Fort, J., Perez-Losada, J., & Isern, N. Fronts from integro-difference equations and persistence effects on the Neolithic. Phys. Rev. E**76**, 031913 (2007).

18. Hewlett, B. S., van de Koppel, J. M. H. & Cavalli-Sforza, L. L. Exploration and mating range of Aka pygmies of the Central African Republic. In L.L. Cavalli-Sforza, ed., African pygmies (Academic Press, Orlando, 1986), pp. 65-80. The data used appear in p.76.
